# Synthesis and Characterization of Magnesium Co-Substituted M-Type Ferrites BaFe_12−*x*−*y*_Mg_*x*_*M*_*y*_O_19_ with *M* = Zr, Hf

**DOI:** 10.3390/ma19122626

**Published:** 2026-06-18

**Authors:** Yanina Mariella Dreer, Ivan Shestov, Deven P. Estes, Rainer Niewa

**Affiliations:** 1 Institute of Inorganic Chemistry, University of Stuttgart, Pfaffenwaldring 55, 70569 Stuttgart, Germany; 2 Institute of Technical Chemistry, University of Stuttgart, Pfaffenwaldring 55, 70569 Stuttgart, Germany; deven.estes@itc.uni-stuttgart.de

**Keywords:** hexaferrites, co-substitution, magnesium, zirconium, hafnium, magnetism, charge compensation

## Abstract

M-type hexaferrites are widely used in magnetic applications, and tailoring their properties via aliovalent substitution requires a detailed understanding of charge compensation and cation distribution. In this work, Mg^2+^/*M*^4+^ (*M* = Zr, Hf) co-substituted BaFe_12_O_19_ was synthesized via Na_2_CO_3_ flux and comprehensively characterized by wavelength-dispersive X-ray spectroscopy, powder and single-crystal X-ray diffraction, Rietveld refinement, X-ray absorption near-edge structure, and magnetic measurements. Increasing substitution levels *x*, *y* in BaFe_12−*x*−*y*_Mg*_x_M_y_*O_19_ result in increasing lattice parameters and decreasing the room-temperature magnetic parameters saturation magnetization, remanence, and coercivity, while remanence and coercivity increase at low temperatures. Secondary phases form for nominal substitution ≥ 1. Zr^4+^ and Hf^4+^ preferentially occupy the 4*f*_2_ site, whereas Mg^2+^ is distributed over multiple sites, as indicated by polyhedral volume analysis. Wavelength-dispersive X-ray spectroscopy confirms homogeneous elemental distribution within individual crystals but reveals significant variation in substitution levels within batches. The maximum degree of substitution for the tetravalent metals was *y* ≈ 1.2–1.7, with lower Mg incorporation of *x* ≈ 0.9–1.1. Charge compensation was found to be partially achieved via vacancy formation, while minor Fe^2+^ contributions cannot be excluded.

## 1. Introduction

Due to their ferrimagnetic properties, hexaferrites are of great interest for applications such as permanent magnets, EMI shielding, or as microwave absorbers [1]. In order to influence the crystallographic and magnetic structure and thus the properties of hexaferrites, a wide range of possible substitutions of iron and barium were studied. For example, Shams et al. proposed Mg/Ti-substituted barium hexaferrites as potential electromagnetic wave absorption materials [2]. This work focuses on the tailoring of the hexaferrite properties through the co-substitution of iron with magnesium and zirconium or hafnium.

In theory, the aliovalent substitution of Fe^3+^ with equal amounts of Mg^2+^ and *M*^4+^ (*M* = Zr, Hf) should preserve the charge neutrality of the ferrite. A similar approach was already pursued by Liu et al. in the synthesis of Sr_1−*x*_Gd*_x_*Fe_12−*x*_Mg*_x_*O_19_, where the additional positive charge introduced by Gd^3+^ at the divalent alkaline earth metal site is compensated by Mg^2+^ at the Fe^3+^ site [3]. Depending on the synthesis route of the desired substituted ferrites, one can observe deviations from the equimolar intake of the co-substituting ions, typically resulting in an increased share of the higher charged ion. For the balance of the resulting excess positive charge, several mechanisms can be envisioned, with the reduction of Fe^3+^ to Fe^2+^ and vacancies at the transition metal sites as the most likely ones [1,4].

**Figure 1:**
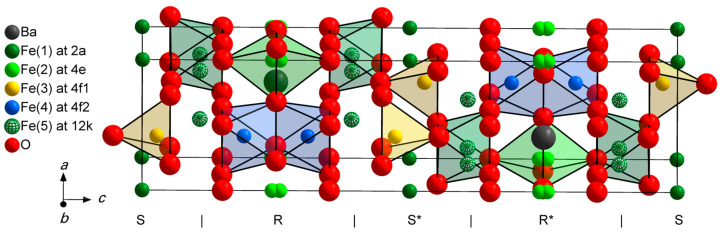
Extended unit cell of BaFe_12_O_19_ with labeled blocks and coordination polyhedra around selected cations.

However, not only is the realization of charge compensation of interest, but also the distribution of ions within the hexaferrite. The crystal structure of BaFe_12_O_19_ (**BaM**), based on close-packed oxygen layers, is shown in Figure 1 and can be divided into R- and S-blocks. The R-block consists of three consecutive hexagonally stacked close-packed oxygen layers, whereas the S-block comprises two cubic close-packed oxygen layers. The stacking sequence of **BaM** can thus be described as RSR*S*, where the asterisk denotes a 180° rotation of the respective block around the *c*-axis. In addition to this block-based description, the structure can be understood in terms of atomic positions and the connectivity of coordination polyhedra surrounding the metal sites. The oxygen atoms occupy five distinct crystallographic Wyckoff positions. Within the central layer of the three hexagonally packed layers in the R-block, one quarter of the oxygen atoms are replaced by barium ions (2*d*), which are coordinated in an anticuboctahedral manner. The iron atoms occupy the voids formed by the stacking of the oxygen layers and are distributed over five crystallographically distinct Wyckoff positions and are coordinated in an octahedral (Fe(1), 2*a*; Fe(4), 4*f*_2_; Fe(5), 12*k*), tetrahedral (Fe(3), 4*f*_1_), and trigonal bipyramidal manner (Fe(2), 4*e*). The latter is more accurately described as a split position with 50% occupancy and a coordination environment closer to tetrahedral than to an ideal trigonal bipyramidal geometry at the 2*b* site.

Liu et al. identified the transition metal sites 4*f*_1_, 4*f*_2_, and 2*b* as preferred positions for magnesium in Sr_1−*x*_Gd*_x_*Fe_12−*x*_Mg*_x_*O_19_ based on Rietveld and Raman data [3]. Sharbati et al. assigned both magnesium and zirconium in SrFe_12−2*x*_Mg*_x_*Zr*_x_*O_19_ preferentially to the 4*f*_1_ site based on magnetic measurements [5]. In contrast, Shams et al. observed a distribution of the substituting ions over the 2*a*, 12*k*, and 2*b* (4*e*) sites in BaFe_9_Mg_1.5_Ti_1.5_O_19_ [2].

Several groups report for various Mg-substituted M-type ferrites that the saturation magnetization *M*_S_ decreases with increasing degree of substitution [3,6,7,8], while conflicting results have been observed for the coercive field *H*_C_ and remanence *M*_R_. Agustiana et al. report an increase in *H*_C_ and *M*_R_ [6], whereas Beheshti et al. observe decreasing values [7], as do Huang et al. and Liu et al. [3,8].

The aim of this work is to investigate the influence of the substitution of iron with magnesium in combination with zirconium or hafnium. The precise composition was analyzed using wavelength-dispersive X-ray spectroscopy (WDX) measurements, and possible charge-compensation mechanisms were examined by combining different methods such as WDX, X-ray absorption near-edge structure (XANES) measurements, and single-crystal X-ray diffraction (SCXRD). A comparison of data obtained from powder X-ray diffraction (PXRD), SCXRD, and WDX is intended to provide insights into the maximum achievable degree of substitution. Furthermore, the magnetic properties of the prepared samples are investigated in order to establish a better correlation between structure and observed properties, in combination with the comprehensive characterization carried out beforehand.

## 2. Materials and Methods

Fifteen hexaferrite samples with the general composition BaFe_12−*x*−*y*_Mg*_x_*Zr*_y_*O_19_ (**MgZr(1)**–**MgZr(15)**) were synthesized using sodium carbonate as a flux, with nominal substitution levels of *x*_nom_ = *y*_nom_ = 0.10–1.50 in increments of 0.10. Appropriate molar amounts of BaCO_3_ (p.a., Carl Roth, Karlsruhe, Germany), Fe_2_O_3_ (99.0%, Riedel de Haën, Seelze, Germany), MgO (p.a., Merck, Darmstadt, Germany), ZrO_2_ (PSZ, Merck), and 25 mol-% Na_2_CO_3_ (p.a., Honeywell, Charlotte, NC, USA) were thoroughly mixed, ground in an agate mortar, and heated in a platinum crucible under air to 1300 °C over a period of ten hours. This temperature was maintained for 36 h, followed by cooling to 500 °C for 60 h. The furnace was then switched off, and the resulting crude product was washed with half-concentrated nitric acid and demineralized water. The obtained products never contained any remains of carbonates, but occasionally minor amounts of platinum from mechanical separation from the crucible surface, according to powder X-ray diffraction (PXRD). In the case of single-phase products, synthesis and post-treatment typically yielded more than 90% of the expected amount of product. For information on by-products of highly substituted samples close or above the maximal degree of substitution, please refer to the individual sections in the manuscript, particularly Section 3.1 (PXRD and lattice parameters).

The BaFe_12−*x*−*y*_Mg*_x_*Hf*_y_*O_19_ series includes eight samples (**MgHf(1)**–**MgHf(8)**) with *x*_nom_ = *y*_nom_ = 0.20–1.60 (0.20 increments). Hafnium was introduced using HfO_2_ (99.9%, Zr ≤ 0.5%, Merck). The synthesis followed the same procedure as for the Mg/Zr-substituted materials. Additionally, an unsubstituted BaFe_12_O_19_ (**BaM**) sample was prepared as a reference.

PXRD measurements were performed on a STADI P diffractometer (STOE & Cie GmbH, Darmstadt, Germany) equipped with Mo-*K*_α1_-radiation (λ_Mo_ = 70.930 pm) and a MYTHEN-1K detector (Dectris, Baden, Switzerland). Rietveld refinements were carried out using the Topas software package (TOPAS-Academic-64, Version V7.21) [9] to determine lattice parameters, average degrees of substitution, and, in the case of multiphase samples, approximate phase fractions. Preferred orientation effects were corrected using a model based on March [10].

Single crystals from all samples were selected and mounted in glass capillaries for single-crystal X-ray diffraction (SCXRD) measurements using a κ-CCD four-circle diffractometer (Bruker-Nonius, Karlsruhe, Germany) with Mo-*K*_α_ radiation (λ_Mo_ = 71.073 pm). Structure solution and refinement were performed with SHELXL-2018/1 [11], while polyhedra volumes were calculated using the Polynator software V1.7.1 [12]. During refinement, full occupancy of all cation sites was assumed.

Crystal compositions were determined by energy- and wavelength-dispersive X-ray spectroscopy (EDX and WDX) using an SX 100 electron microprobe analyzer (Cameca, Gennevilliers, France) equipped with a scanning electron microscope (SEM). Multiple crystals from each sample, including those used for SCXRD, were analyzed. Prior to measurement, samples were coated with a thin carbon layer to prevent charging. Quantification was carried out using baryte BaSO_4_ (for Ba), hematite Fe_2_O_3_ (for Fe), periclase MgO (for Mg), zirconium Zr (for Zr), and hafnium Hf (for Hf) as standards.

Magnetic measurements on single-phase powder samples were performed using a SQUID magnetometer MPMS3 (Quantum Design, Pfungstadt, Germany). For hysteresis measurements at −270 °C and 27 °C, approximately 10–20 mg of ground sample was packed into a gelatin capsule with cotton wool, attached to a plastic straw, and inserted into the instrument.

To further investigate the electronic states of iron, X-ray absorption spectroscopy (XAS) was conducted at the Fe K-edge (7112 eV) using an easyXAFS300+ spectrometer (easyXAFS, Renton, WA, USA) equipped with a KETEK AXAS silicon drift detector and an X-ray source with a silver anode operated at 20 kV and 12 mA, utilizing the (440) harmonic of a spherically bent-channel cut Si(110) crystal analyzer for energy selection. Data were collected with an acquisition time of 15 s per point, and each spectrum represents the average of seven scans. Samples were prepared by grinding 15–20 mg of microcrystalline powder in an agate mortar and mounting it between X-ray amorphous adhesive tape layers. Data analysis was performed using the Demeter software package V0.9.26 [13] and oxidation states were evaluated by comparison with reference compounds (FeCl_2_∙4H_2_O and BaFe_12_O_19_) measured under identical conditions. Energy calibration was carried out using the absorption edge of an iron foil.

## 3. Results and Discussion

Samples of BaFe_12−*x*−*y*_Mg*_x_M_y_*O_19_ with *M* = Zr, Hf were synthesized from a sodium carbonate flux. Microcrystalline powders of the ground samples were investigated via powder X-ray diffraction (PXRD), magnetization, and X-ray absorption spectroscopy (XAS) measurements. Additionally, several single crystals were selected for single-crystal X-ray diffraction (SCXRD) and wavelength-dispersive X-ray spectroscopy (WDX). The latter techniques reveal that, within a given batch, the actual substitution level of individual crystals can vary significantly. Therefore, when interpreting the results, it is important to distinguish between measurements on single crystals and those on powder samples.

### 3.1. PXRD and Lattice Parameters

**Figure 2:**
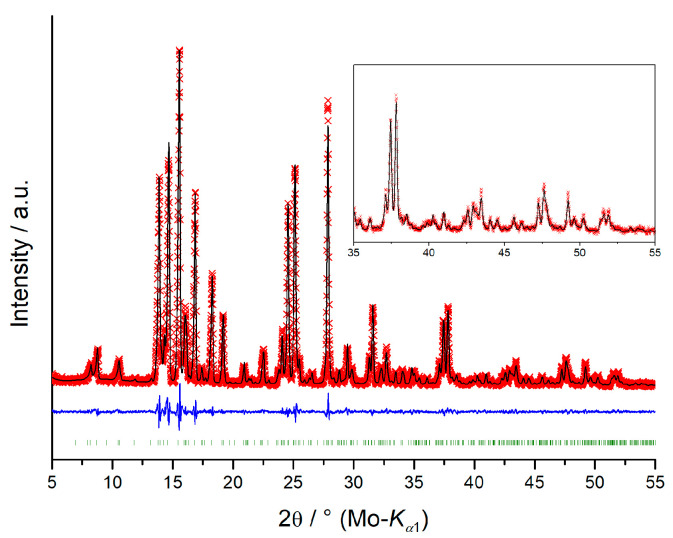
PXRD measurement of sample **MgZr(4)** (red) with Rietveld refinement (black), difference curve (blue), and possible Bragg positions (BaM—green). Inset: Enlarged (×2) range from 35–55°; gof 1.33.

To verify phase purity and determine phase fractions in the presence of secondary phases, powder X-ray diffraction patterns were recorded for microcrystalline powders from all batches after post-treatment, as described in Section 2. For a more detailed analysis of the samples, Rietveld refinements were subsequently carried out on all PXRD data. Figure 2 shows, as a representative example, the Rietveld refinement of sample **MgZr(4)**; all other refinements can be found in the Supporting Information (Figures S1–S22). These data indicate that at high nominal degrees of substitution *x*_nom,Mg/Zr_ ≥ 1.10 and *x*_nom,Mg/Hf_ ≥ 1.00, an increased presence of secondary phases is observed, either in the form of the perovskites BaZrO_3_ and BaHfO_3_ or as remnants of starting materials that did not react during synthesis (MgO, Fe_2_O_3_, HfO_2_). In addition, small amounts of platinum can be detected in some samples regardless of the substitution level, which can be attributed to the mechanical removal of the product from the platinum crucible. Single-phase samples were obtained for BaFe_12_O_19_ (**BaM**), BaFe_12−*x*−*y*_Mg*_x_*Zr*_y_*O_19_ with 0.10 ≤ *x* ≤ 0.90 (**MgZr(1)**–**MgZr(9)**) and BaFe_12−*x*−*y*_Mg*_x_*Hf*_y_*O_19_ with 0.20 ≤ *x* ≤ 0.80 (**MgHf(1)**–**MgHf(4)**).

**Figure 3:**
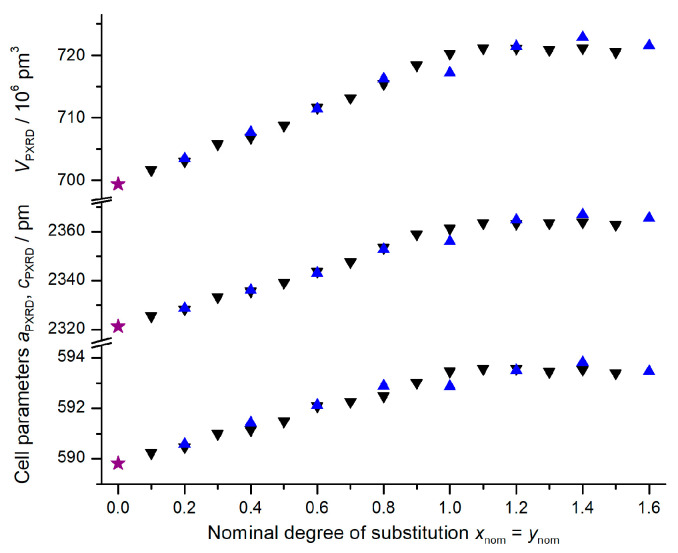
Unit cell parameters *a* and *c* and unit cell volume *V* from Rietveld refinement for **BaM** (purple), and Mg/Zr- (black), and Mg/Hf-substituted samples (blue) versus the nominal degree of substitution *x*_nom_ = *y*_nom_. All error bars are significantly smaller than the displayed symbols.

**Figure 4:**
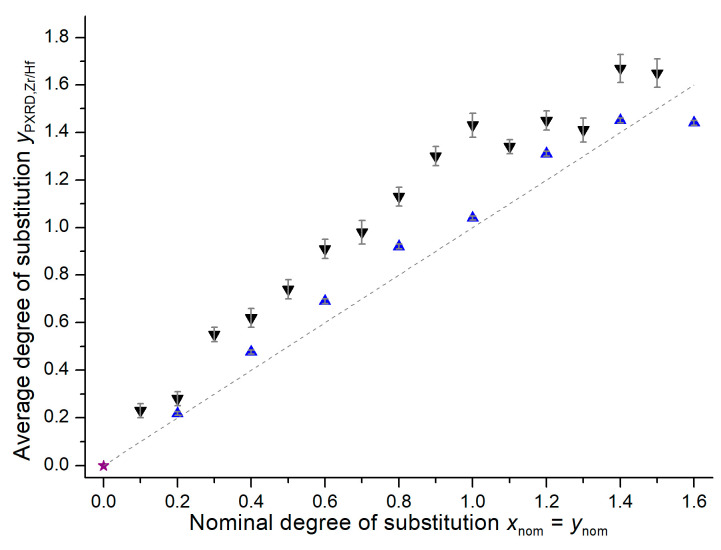
Average degree of substitution *y*_PXRD,Zr_ (black) and *y*_PXRD,Hf_ (blue), including error bars for the M-type hexaferrite obtained from Rietveld refinements versus the nominal degree of substitution (**BaM** purple). The dashed line represents the ideal relationship *y*_nom_ = *y*_PXRD_ and is intended as a guide to the eye.

The unit cell parameters of the target compounds obtained from the Rietveld refinements are listed in Table S1 and plotted against the nominal degree of substitution in Figure 3. For both substitution series, a nearly linear increase in the unit cell parameters and the unit cell volume can be observed up to *x*_nom_ = 0.80. A closer look at the effective ionic radii [14] of the relevant metal ions considered here (Table S2) shows that this behavior is indeed expected for a successful substitution of Fe^3+^ with the larger ions of Mg^2+^, Zr^4+^, and Hf^4+^. For higher degrees of nominal substitution, the unit cell parameters of the Mg/Hf-substituted ferrites gradually approach a plateau, which indicates that the maximum substitution level has been reached. The unit cell parameters of the Mg/Zr-substituted ferrites also reach a plateau at higher substitution levels, although only above a nominal substitution level of approximately *x*_nom,Mg/Zr_ = 1.00. During the Rietveld refinements, mixed occupancy of the Fe(4) site (4*f*_2_) with zirconium or hafnium was taken into account, and the resulting degree of substitution *y*_PXRD,Zr/Hf_ is shown in Figure 4 in comparison with *y*_nom_. In contrast, the substitution of iron with magnesium was neglected in the refinements carried out here. A review of the literature on Mg-substituted hexaferrites shows that the localization of magnesium in the structure appears difficult using PXRD, despite the significantly lower scattering power of Mg compared to iron. While Sharbati et al. identified the tetrahedrally coordinated Fe(3) site (4*f*_1_) as the preferred position for Mg in SrFe_12−2*x*_Mg*_x_*Zr*_x_*O_19_ based on magnetic measurements [5], Davoodi et al. reported a preference for the octahedrally coordinated iron sites (Fe(1) (2*a*), Fe(4) (4*f*_2_), Fe(5) (12*k*)), particularly the Fe(4) site (4*f*_2_) for Mg in SrFe_12−*x*_(Sn_0.5_Mg_0.5_)*_x_*O_19_ [15]. Liu et al., combining PXRD, Raman, and magnetic data, identified the trigonal-bipyramidal Fe(2) site (2*b* or 4*e*) and the octahedrally coordinated Fe(4) site (4*f*_2_) as preferred positions for magnesium in Sr_1−*x*_Gd*_x_*Fe_12−*x*_Mg*_x_*O_19_, while also finding significant occupancy at the tetrahedrally coordinated Fe(3) site (4*f*_1_). From PXRD data, a certain fraction of magnesium could be refined at each of the five crystallographic iron sites [3]. Based on the powder X-ray diffraction data available here for BaFe_12−*x*−*y*_Mg*_x_*Zr*_y_*O_19_ and BaFe_12−*x*−*y*_Mg*_x_*Hf*_y_*O_19_, no preferred site for magnesium could be identified. The choice of the Fe(4) site (4*f*_2_) for mixed occupancy with tetravalent cations and the possible localization of magnesium ions are discussed in more detail in Section 3.5.

Figure 4 shows that the average degree of substitution of Zr (*y*_PXRD,Zr_) and Hf (*y*_PXRD,Hf_) obtained from Rietveld refinements is almost always higher than the nominally targeted value. One possible reason for this could be that, during the synthesis of the ferrites, secondary phases are formed that do not contain zirconium or hafnium but alter the molar ratios of the starting material available for reaction within the flux, thereby leading to a higher degree of substitution at lower product yield. These secondary phases may not be detectable after post-synthetic treatment with half-concentrated nitric acid and demineralized water, meaning that some possible by-products had already been removed.

### 3.2. Magnetic Properties

**Figure 5:**
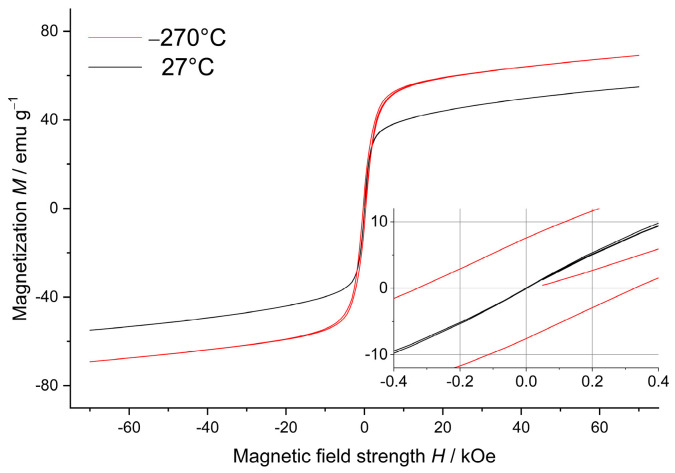
Hysteresis curve of exemplary sample **MgZr(9)** measured at −270 °C (red) and 27 °C (black). Inset: Enlargement of the area around the origin. For full measurements of all discussed samples, please compare Figures S23–S26.

**Figure 6:**
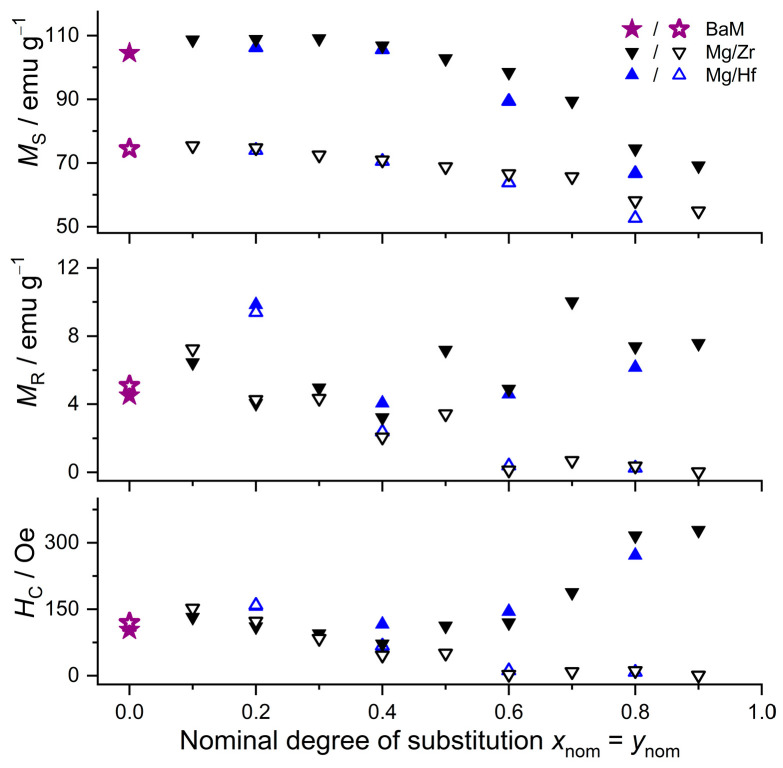
Magnetic parameters of **MgZr(1)**–**MgZr(9)** (black) and **MgHf(1)**–**MgHf(4)** (blue) in comparison with unsubstituted **BaM** (purple) plotted against the nominal degree of substitution *x*_nom_ = *y*_nom_. The filled symbols represent the measurement data at −270 °C, while the open symbols indicate the measurements at 27 °C.

To gain insights into the magnetic properties of the Mg co-substituted ferrites, hysteresis curves of all single-phase samples (**BaM**, **MgZr(1)**–**MgZr(9)**, **MgHf(1)**–**MgHf(4)**) were recorded at −270 °C and 27 °C. Figure 5 shows, as a representative example, the hysteresis curves of sample **MgZr(9)**; the measurement curves of the other samples can be found in Figures S23–S26, and all values are summarized in Table S3. The values of the coercive field strength *H*_C_, the remanence *M*_R_, and the saturation magnetization *M*_S_ obtained from the measurements are presented graphically in Figure 6.

The expected change in magnetic properties with increasing degree of substitution depends on the crystallographic site at which iron ions carrying a magnetic moment are replaced by diamagnetic ions. From Figure 6, it can be seen that the saturation magnetization *M*_S_ remains nearly constant for both Mg/Zr and Mg/Hf at 27 °C and −270 °C up to *x*_nom_ ≈ 0.4 and then decreases sharply for *x*_nom_ ≥ 0.5. This behavior is in good agreement with the results published by Beheshti et al. for BaFe_12−2*x*_Mg*_x_*Zr*_x_*O_19_ with 0.25 ≤ *x* ≤ 1.5 measured at room temperature [7]. Furthermore, Beheshti et al. observed decreasing values for remanence and coercive field strength at room temperature at higher *x* [7]. Considering first the Mg/Zr-substituted samples, a slight increase in *M*_R_ and *H*_C_ can be observed at *x* = 0.1. For *x* = 0.2–0.3, the values return to their initial level. At 27 °C, decreasing values of remanence and coercive field strength are observed for *x* ≥ 0.4, whereas at −270 °C they increase. The remanence shows upward outliers at *x*_nom_ = 0.5 and 0.7. The Mg/Hf-substituted ferrites exhibit the same behavior as the Mg/Zr series with respect to coercive field strength. However, for *M*_R_, a strong increase is observed at *x*_nom_ = 0.2 independent of temperature. At *x*_nom_ = 0.4, the remanence returns to its initial value and then decreases sharply at 27 °C, while at −270 °C it increases again, though it does not reach its previous level. The increasingly disrupted super-exchange interactions, combined with a growing number of magnetic domains, can explain the increase in remanence and coercive field strength with substitution observed at low temperatures for both substitution series. Due to the low thermal energy at these temperatures and therefore reduced thermal vibrations of the ions, the domains likely have to be demagnetized individually, requiring progressively higher field strengths than at 27 °C. Similarly, the residual magnetization can be maintained more effectively in the absence of thermally induced vibrations. Similar observations were made in previous works for the system BaFe_12−*x*−*y*_Mn*_x_*Ti*_y_*O_19_ [16] and the substitution series of BaFe_12−*x*−*y*_Zn*_x_M_y_*O_19_ with *M* = Sn, Zr, Hf [17].

### 3.3. Composition Determination via WDX

**Figure 7:**
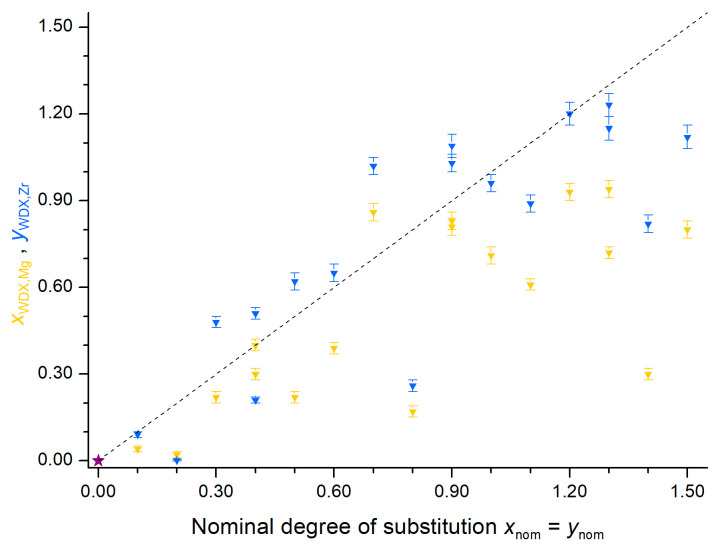
Degree of substitution obtained from WDX for zirconium (blue) and magnesium (yellow) versus nominal degree of substitution for BaFe_12−*x*−*y*_Mg*_x_*Zr*_y_*O_19_ (**BaM** purple). The dashed line represents the ideal progression for *x*_WDX_, *y*_WDX_ = *x*_nom_ = *y*_nom_, and is intended as a guide for the eye.

**Figure 8:**
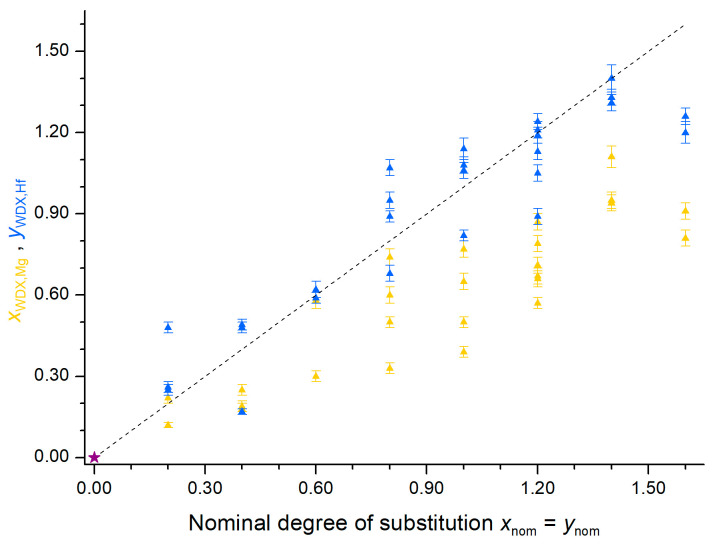
Degree of substitution obtained from WDX for hafnium (blue) and magnesium (yellow) versus nominal degree of substitution for BaFe_12−*x*−*y*_Mg*_x_*Hf*_y_*O_19_ (**BaM** purple). The dashed line represents the ideal progression for *x*_WDX_, *y*_WDX_ = *x*_nom_ = *y*_nom_, and is intended as a guide for the eye.

To ensure improved comparability of the results, the identical crystals were analyzed for the WDX measurements discussed in this section, as were used for SCXRD (Section 3.5). In addition, several further crystals were selected and examined using the electron microprobe; however, not all were suitable for SCXRD. These crystals are labeled with the index “r” followed by a consecutive number for each sample. Depending on crystal size and quality, 4–10 distinct measurement points were analyzed per crystal. Quantification of the results was performed using the standard materials listed in Section 2. The oxygen content was calculated under the assumption that the cations are present as Ba^2+^, Fe^3+^, Mg^2+^, Zr^4+^, and Hf^4+^. The average mass fractions $\bar{w}$ of the elements, calculated from the individual measurement points, are summarized in Table S4 for BaFe_12−*x*−*y*_Mg*_x_*Zr*_y_*O_19_ and in Table S5 for BaFe_12−*x*−*y*_Mg*_x_*Hf*_y_*O_19_. Assuming a fully occupied oxygen sublattice (i.e., no oxygen vacancies), the compositions were normalized to the oxygen content, and the corresponding empirical formulas were derived. Subtracting the combined fractions of iron, magnesium, and zirconium/hafnium from the full occupancy of the transition metal sites allows the calculation of the theoretical vacancy concentration □_WDX_ for each crystal. These values, together with the iron, magnesium, and zirconium or hafnium contents, are listed in Tables S6 and S7. Furthermore, the magnesium and zirconium or hafnium contents obtained from the WDX measurements are plotted as a function of the nominal degree of substitution in Figure 7 and Figure 8. Typically, good data quality is indicated by mass-fraction sums close to 100%; however, some data suffer from the accumulation of errors for the individual measurements of elements, the indirect quantification of oxygen using assumptions about valence states as described above, and measurement effects, e.g., nonparallel surface and surface roughness.

**Figure 9:**
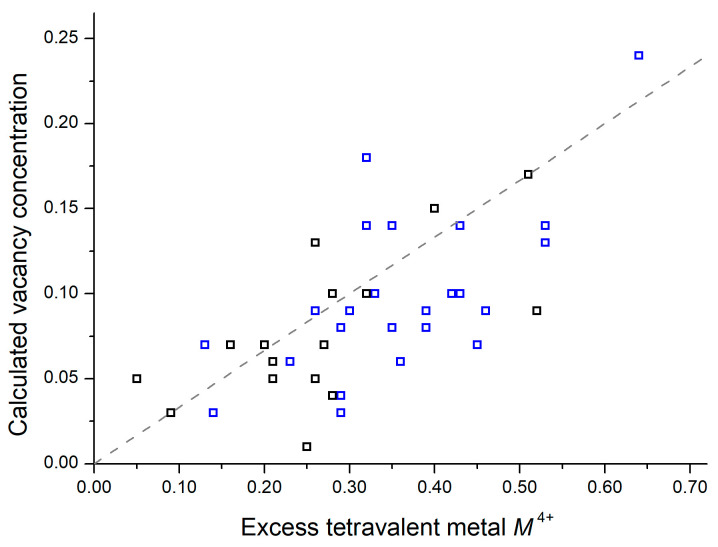
Vacancy concentrations calculated from WDX measurements plotted against the excess (*y_M_*^4+^ − *x*_Mg_^2+^) of the tetravalent metal (black—Zr; blue—Hf). The dashed line represents the ideal progression for exclusive charge balance by formation of vacancies and is intended as a guide for the eye.

Based on the data presented here, several conclusions can be drawn for Mg-substituted hexaferrites of the type BaFe_12−2*x*_Mg*_x_M_x_*O_19_ with *M* = Zr, Hf. First, it is evident that the tetravalent metal is preferentially incorporated into the ferrite structure compared to divalent magnesium, with only a few exceptions. As a consequence, the general composition of these compounds is more accurately described by BaFe_12−*x*−*y*_Mg*_x_M_y_*O_19_, with *y* ≥ *x*. Furthermore, it becomes apparent that the degree of substitution of individual crystals within a single batch can vary significantly. As a result, individual crystals may exhibit substitution levels exceeding the nominal target composition. For the comparison of trends in composition, substitution degree, or unit cell parameters obtained by different methods, it is essential to distinguish whether the discussion refers to single-crystal data or to values averaged over the bulk powder. For instance, the unit cell parameters obtained from Rietveld refinement of PXRD data (Section 3.1) suggested a substitution limit of approximately *x*_nom_ = *y*_nom_ = 0.8–1.0. In contrast, WDX measurements revealed maximum substitution levels of *x*_WDX,Mg_ = 0.94(3) and *y*_WDX,Zr_ = 1.23(4) for Mg/Zr-substituted ferrites, whereas even higher values of up to *x*_WDX,Mg_ = 1.11(4) and *y*_WDX,Hf_ = 1.40(5) were observed for Mg/Hf-substituted systems. In both cases, an excess of the tetravalent cation is present, resulting in a positive charge imbalance that requires compensation. As noted above, this subsection primarily addresses the possible formation of vacancies on the transition metal sites. The vacancy concentrations listed in Tables S6 and S7 are, in most cases, within the uncertainty of the compositional analysis and should therefore be interpreted with caution. Negative values of the vacancy concentration are physically meaningless and are attributed to measurement uncertainties and errors introduced during normalization. In such cases, all transition metal sites are assumed to be fully occupied. If charge compensation were to occur exclusively via vacancy formation, each additional tetravalent ion would need to be balanced by one-third of a vacancy. This relationship is illustrated by the dashed line in Figure 9. The vacancy concentrations derived from the WDX data are also shown in this figure. Although they do not coincide with the expected linear relationship, a general trend can be observed: □_WDX_ increases with increasing excess of the tetravalent cation for both the Mg/Zr and Mg/Hf series. This behavior may indicate that vacancy formation contributes, at least in part, to charge compensation in Mg-substituted ferrites.

**Figure 10:**
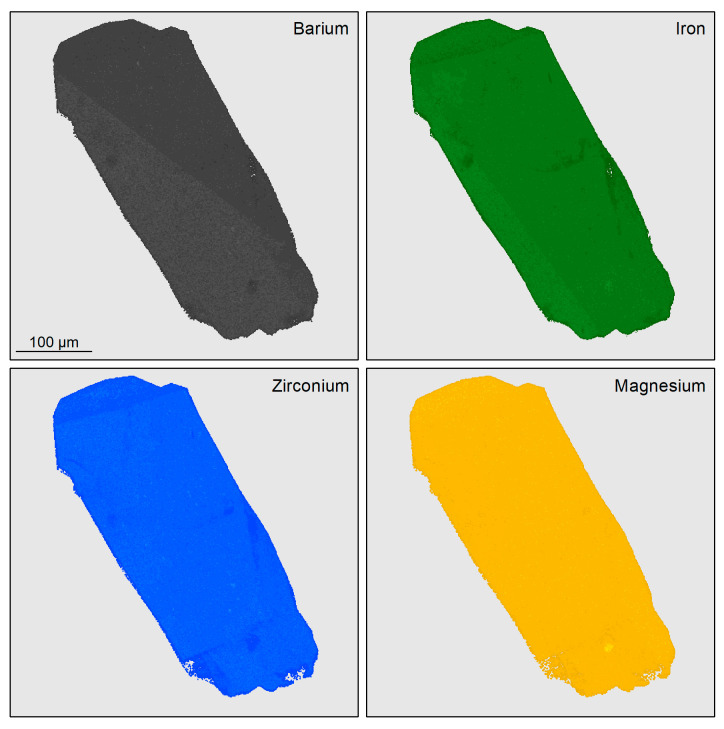
EDX maps of **MgZr(12)**_sc1_ for barium, iron, zirconium, and magnesium.

**Figure 11:**
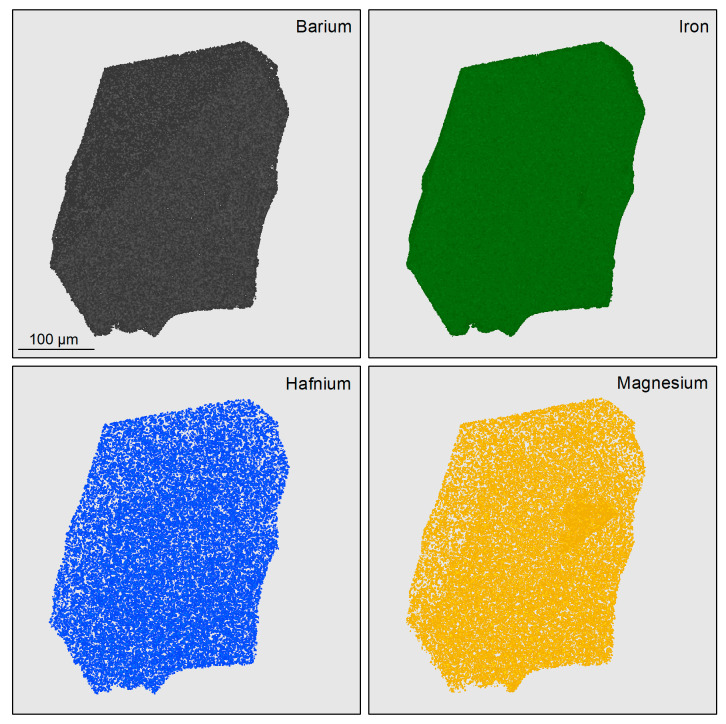
EDX maps of **MgHf(1)**_sc1_ for barium, iron, hafnium, and magnesium.

Another important aspect is the distribution of the elements within the crystal and across its surface. Therefore, one representative crystal was selected for each substitution series for elemental mapping in addition to the WDX measurements. As shown in Figure 10 and Figure 11, all elements are homogeneously distributed across the surface, and no agglomeration of the substituting metals is observed. It can thus be assumed that the composition determined by WDX corresponds to the average empirical formula of the crystal and that any chosen measurement point on the crystal exhibits essentially the same composition.

### 3.4. XAS Investigations

Based on the observation discussed in the previous section that a higher proportion of the tetravalent cation than of the divalent cation was incorporated in the ferrite structure of BaFe_12−*x*−*y*_Mg*_x_M_y_*O_19_, the valence states of iron are examined in more detail. It is assumed that both zirconium and hafnium are unequivocally present in the +IV oxidation state under the synthesis conditions, while magnesium occurs exclusively as Mg^2+^. Thus, the additional positive charge of the tetravalent cation in BaFe_12−*x*−*y*_Mg*_x_M_y_*O_19_ with *M* = Zr, Hf, and *y* > *x* must be compensated. Various mechanisms for charge balance are possible, of which two are considered in more detail here: formation of vacancies on the transition metal sites (see Section 3.3) and partial reduction of Fe^3+^ to Fe^2+^. Naturally, a combination of both processes is also possible, as it was observed in previous studies of Ti-substituted ferrites BaFe_12−*x*_Ti*_x_*O_19_ at high degrees of substitution [4]. In contrast, Mn-substituted ferrites BaFe_12−*x*_Mn*_x_*O_19_ realize charge balance by the simultaneous presence of Mn^2+^, Mn^3+^, and Mn^4+^, while no indications for partial reduction of Fe^3+^ or vacancy formation were observed [18].

**Figure 12:**
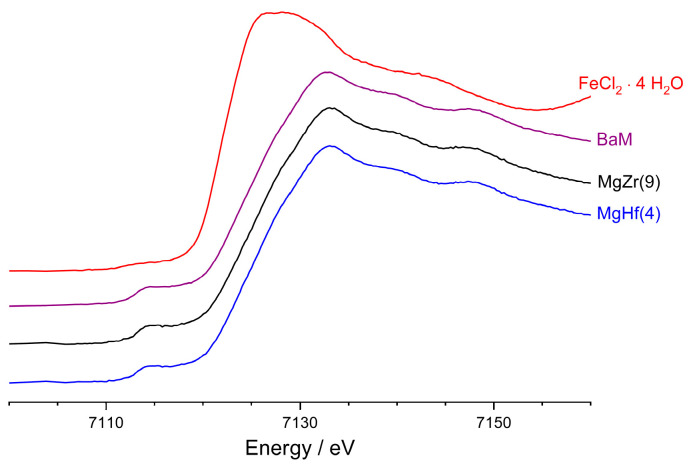
Fe K-edge XANES spectra of **MgZr(9)** (black) and **MgHf(4)** (blue) compared to the spectra of FeCl_2_∙4 H_2_O (red) and **BaM** (purple).

To obtain indications of the possible presence of Fe^2+^ species in BaFe_12−*x*−*y*_Mg*_x_M_y_*O_19_, X-ray absorption near-edge structure (XANES) spectra at the Fe K-edge were recorded for the two most highly substituted yet still single-phase samples of the Mg/Zr and Mg/Hf substitution series, as these should exhibit the highest fraction of Fe^2+^ and therefore provide the most reliable data. Figure 12 shows the XANES spectra of the samples **MgZr(9)** and **MgHf(4)** together with the references. Even qualitatively, it is evident from the figure that the spectra of both samples are very similar to the curve profile of **BaM**. This impression is confirmed when attempting to represent the sample spectra as linear combinations of the reference spectra. It becomes clear that the Fe^2+^ fraction must lie at the lower end of the detection limit, which is approximately 5%, indicating only a compensation by Fe^2+^ to a small extent.

### 3.5. Influence of the Substitution on the Crystal Structure

**Figure 13:**
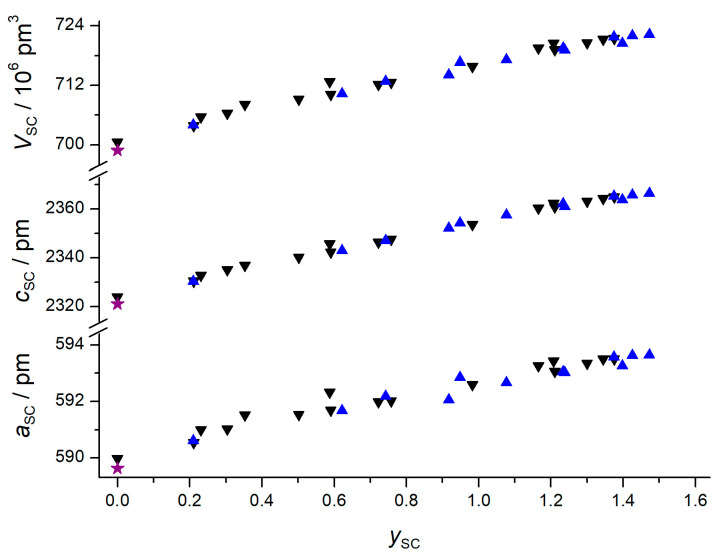
Unit cell parameters *a*_SC_ and *c*_SC_ and unit cell volume *V*_SC_, derived from SCXRD data for **BaM** (purple), Mg/Zr- (black), and Mg/Hf-substituted samples (blue), plotted as a function of the degree of substitution *y*_SC_ of the tetravalent cations obtained from the corresponding structure refinements.

As introduced in Section 3.3, all crystals labeled with the index “sc” were additionally investigated by SCXRD. This enables a direct comparison of the compositions obtained from the different analytical methods and contributes significantly to the overall interpretation of the results. In addition, several further single crystals were selected for SCXRD. However, due to their morphology, these were not suitable for investigation by electron microprobe or, in some cases, could not be successfully transferred from the capillary to the adhesive pad. Figure 13 shows the evolution of the unit cell parameters as a function of the degree of substitution *y*_SC_ of the tetravalent metals, as determined from the structure refinement. As already observed from the PXRD data, a nearly linear increase of the lattice parameters with increasing substitution level is evident. The values of *a*_SC_, *c*_SC_, and *V*_SC_ for the single crystals fall within the same range as the averaged values obtained from Rietveld refinements of PXRD data. However, in contrast to the unit cell parameters derived from Rietveld refinements, no transition to a plateau is observed at this stage.

A closer look at the degree of substitution *y_M_*^4+^ further reveals variations between the different analytical methods. For Mg/Zr-substituted ferrites, maximum substitution levels of *y*_WDX,Zr,max_ = 1.23(4) from WDX measurements, *y*_SC,Zr,max_ = 1.37(4) from structure refinement of SCXRD data, and *y*_PXRD,Zr,max_ = 1.67(6) from Rietveld refinement of PXRD data are obtained. The observed spread in values can be explained, on the one hand, by the fact that SCXRD and WDX measurements probe individual crystals, whereas PXRD data represent an average over a larger quantity of ground microcrystalline powder. On the other hand, the determination of substitution levels from Rietveld refinement of powder X-ray diffraction data is generally more prone to errors, as it relies primarily on changes in the intensities of selected reflections compared to the unsubstituted ferrite in combination with a typically high number of refinement parameters. These intensities, however, can be significantly affected by preferred orientation of the crystallites during measurement. Furthermore, since highly substituted crystals typically exhibit poorer crystal growth, it is possible that neither WDX nor SCXRD measurements captured the most highly substituted specimens, as these may exist only as microcrystalline powder or poly-crystalline material, which is not suitable for these techniques. In addition, magnesium was not considered in the compositional analysis derived from PXRD and SCXRD, which may also lead to deviations in the estimated zirconium content.

In the series of Mg/Hf-substituted ferrites, the variation in the maximum measured substitution level is significantly smaller with values of *y*_WDX,Hf,max_ = 1.40(5), *y*_SC,Hf,max_ = 1.47(1), and *y*_PXRD,Hf,max_ = 1.45(1). A possible explanation for this finding is the substantially larger difference in scattering power between iron and hafnium compared to that between iron and zirconium, allowing the hafnium content to be determined more accurately by X-ray diffraction-based methods. The substitution levels obtained from WDX measurements are considered the most reliable, despite the approximations involved in the evaluation, as, in contrast to the XRD refinements, magnesium is explicitly accounted for. The observation that the zirconium content derived from XRD data exhibits greater variability than the hafnium content may indicate that varying amounts of magnesium and zirconium jointly occupy the Fe(4) site (4*f*_2_). This would significantly complicate an accurate determination of the zirconium content via XRD and simultaneously explain why no occupancy < 1 is observed at other iron sites (see Section 3.1), despite the lower electron density of magnesium. Accordingly, for hafnium-substituted ferrites, it is assumed that magnesium is present only in minor amounts on the Fe(4) site (4*f*_2_), as otherwise similar variations in the degree of substitution as observed for zirconium would be expected.

**Figure 14:**
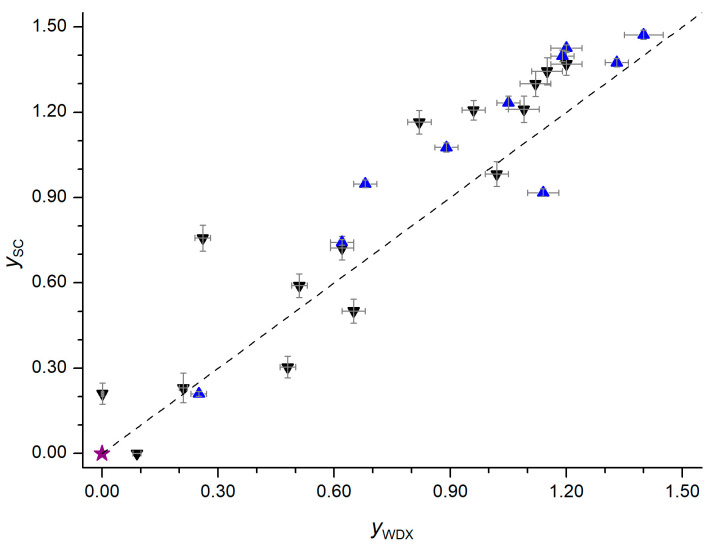
Substitution levels of the tetravalent cations zirconium (black) and hafnium (blue) refined from SCXRD data, compared with the corresponding degree of substitution obtained from WDX measurements on the same crystals (**BaM** purple). The dashed line serves as a guide to the eye for the ideal correlation where *y*_SC_ = *y*_WDX_.

A comparison of the *y*_Zr/Hf_ values obtained from WDX and SCXRD for crystals analyzed by both methods is shown in Figure 14. The same trend as observed for the maximum degrees of substitution is evident, namely that in most cases *y*_SC_ > *y*_WDX_.

**Figure 15:**
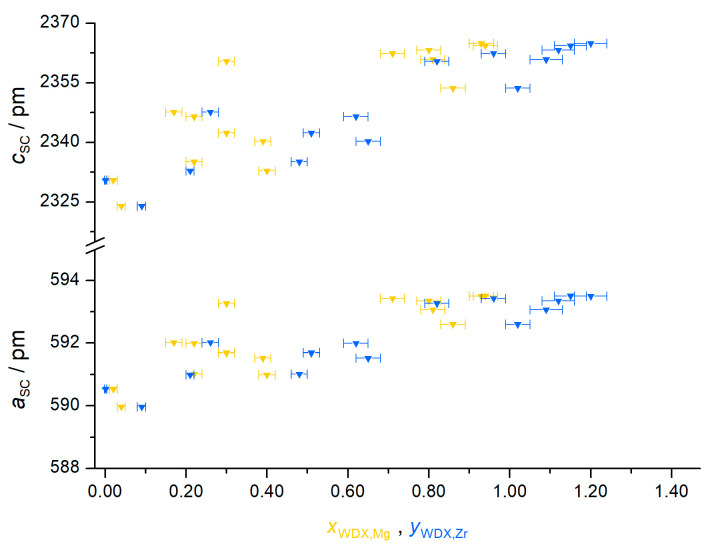
Unit cell parameters *a*_SC_ and *c*_SC_ derived from SCXRD data plotted as a function of the substitution levels *x*_WDX,Mg_ (yellow), and *y*_WDX,Zr_ (blue) obtained from WDX measurements. In total, four data points correspond to each individual crystal; yellow and blue points at the same ordinate value represent the two lattice parameters of a given crystal. The uncertainties of the lattice parameters are smaller than the symbol size and are therefore not shown.

**Figure 16:**
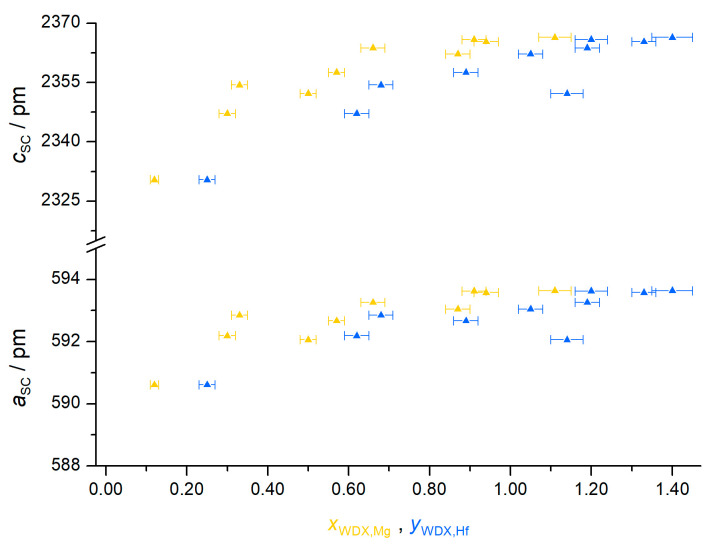
Unit cell parameters *a*_SC_ and *c*_SC_ derived from SCXRD data plotted as a function of the substitution levels *x*_WDX,Mg_ (yellow), and *y*_WDX,Hf_ (blue) obtained from WDX measurements. In total, four data points correspond to each individual crystal; yellow and blue points at the same ordinate value represent the two lattice parameters of a given crystal. The uncertainties of the lattice parameters are smaller than the symbol size and are therefore not shown.

When the unit cell parameters of the single crystals are plotted against the substitution levels of magnesium and zirconium determined by WDX (Figure 15) and hafnium (Figure 16), a clear flattening of the increase in lattice parameters can again be observed. This behavior supports the assumption that the maximum degree of substitution has been reached. In each figure, four data points correspond to a single crystal, comprising the values of the *a*- and *c*-axes in relation to the degrees of substitution *x*_WDX,Mg_, and *y*_WDX,Zr/Hf_.

**Figure 17:**
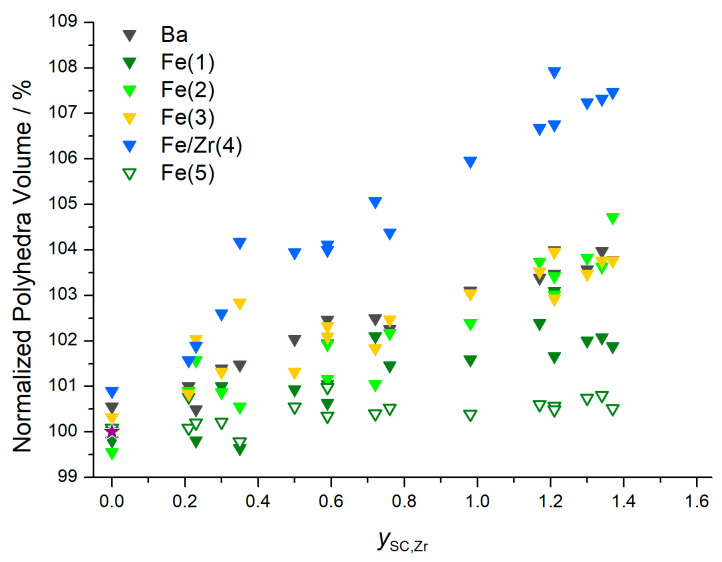
Normalized polyhedra volumes versus degree of substitution *y*_SC,Zr_ obtained from SCXRD refinement for Mg/Zr-substituted M-type ferrites (**BaM** purple).

**Figure 18:**
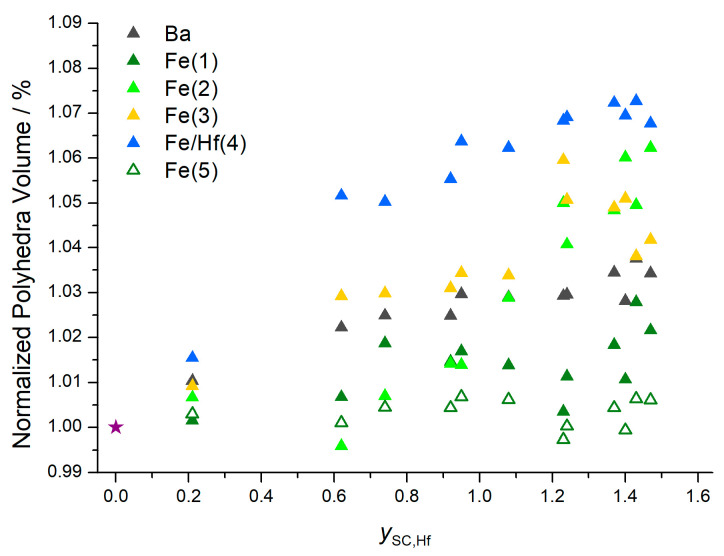
Normalized polyhedra volumes versus degree of substitution *y*_SC,Hf_ obtained from SCXRD refinement for Mg/Hf-substituted M-type ferrites (**BaM** purple).

During the structure refinement of the SCXRD data, the Fe(4) site (4*f*_2_) was unambiguously identified as the preferred site for mixed occupancy with zirconium and hafnium. Even at high substitution levels, incorporation into the M-type crystal structure occurred, within experimental uncertainty, exclusively at the Fe(4) site (4*f*_2_). To further substantiate this observation and to gain insight into the distribution of magnesium, the individual polyhedra volumes are examined in the following. The volumes were calculated using the software Polynator [12] and subsequently normalized to the values of the unsubstituted ferrite. The resulting relative volumes are shown for Mg/Zr-substituted ferrites in Figure 17 and for Mg/Hf-substituted ferrites in Figure 18. The plots are given as a function of the degree of substitution of the tetravalent metal *y*_SC,Zr/Hf_, obtained from SCXRD data. For the Mg/Zr-substituted ferrites (Figure 17), a clear increase in the polyhedra volumes of the octahedrally coordinated Fe(4) site (4*f*_2_) is observed with increasing substitution level, reaching up to 7.5%. In addition, the volumes of the trigonal bipyramid surrounding Fe(2) (4*e*) and the anticuboctahedron around Ba both increase by approximately 3–4%. This is likely not due to direct substitution, but rather to the fact that both Ba and Fe(2), together with Fe(4), are located within the R-block of the M-type structure. Consequently, an expansion of the Fe(4) coordination environment also induces, to some extent, an expansion of the neighboring polyhedra. However, the volume of the tetrahedron around Fe(3) (4*f*_1_) also increases by nearly 4%. This change can no longer be attributed solely to zirconium substitution at the Fe(4) site, as the Fe(3) site and its associated oxygen polyhedron are located within the S-block of the ferrite and should therefore remain unchanged or exhibit only minor variations due to the expansion of the R-block. A possible explanation for the increase in the Fe(3) polyhedra volume is the partial accumulation of magnesium at this site. With an effective ionic radius of 57 pm, Mg^2+^ is significantly larger than Fe^3+^ (49 pm, *CN* 4, high spin; see Table S2) [14]. Sharbati et al. [5] also reported a preferential occupation of the 4*f*_1_ site by magnesium, although zirconium was likewise found at this position. Their interpretation was based on the observed magnetic behavior of the samples, which were synthesized via a sol-gel process. Since no indication of zirconium substitution at the Fe(3) site (4*f*_1_) was found in the present datasets, this is taken as evidence that the observed volume increase is indeed caused by magnesium substitution. On the other hand, refinement of the occupancy factor of Fe(3) did not reveal any significant iron deficiency, suggesting that magnesium is not exclusively located at this site but rather distributed over several crystallographic positions. Given that Mg^2+^ and Zr^4+^ have identical effective ionic radii of 72 pm in six-fold coordination, it is reasonable to assume that part of the magnesium substitutes together with zirconium at the Fe(4) site in the R-block. The Fe(1) site (2*a*), also located in the S-block, exhibits an increase in polyhedron volume of approximately 2%, which is about half of that observed for Fe(3) (4*f*_1_), corresponding to the same ratio as found for Fe(4) relative to Fe(2)/Ba. This suggests that the expansion of the Fe(1) octahedra is largely a consequence of the expansion of the Fe(3) tetrahedra. However, minor contributions from Mg^2+^ (72 pm, *CN* 6) and/or Zr^4+^ (72 pm, *CN* 6) cannot be excluded. Likewise, the presence of small amounts of Fe^2+^ (78 pm, *CN* 6, high spin) at the octahedrally coordinated Fe(1) and Fe(4) sites may also contribute. In contrast, the octahedrally coordinated Fe(5) site (12*k*) shows only a minor volume change of 0.5% and can therefore be excluded as a preferred site for substitution.

The Mg/Hf-substituted ferrites shown in Figure 18 likewise exhibit an increase in the volume of the octahedra surrounding Fe(4) (4*f*_2_) of up to 7.5%, which results from the substitution of Fe^3+^ (64.5 pm, *CN* 6, high spin) by Hf^4+^ (71 pm, *CN* 6). Analogous to the observations for Mg/Zr-substituted ferrites, an expansion of the anticuboctahedra surrounding barium by approximately 3.5–4% is observed. This increase cannot be attributed to direct substitution, but rather to the expansion of the face-sharing octahedra doubles around Fe(4) (4*f*_2_) that share faces with the anticuboctahedron. In contrast to the aforementioned series, the trigonal bipyramids surrounding Fe(2) (4*e*) show an increase in volume of up to 6%. At higher Mg contents *x*_Mg_, this may indicate a minor degree of substitution at the Fe(2) site, leading to a stronger expansion of the coordination polyhedra (Mg^2+^: 66 pm, *CN* 5; Fe^3+^: 58 pm, *CN* 5) than would be expected solely from the neighboring substitution at the Fe(4) site. Similarly, the polyhedral volume of the tetrahedra surrounding Fe(3) (4*f*_1_) increases by approximately 5–6%, suggesting partial localization of magnesium at this site. Liu et al. reported a preferred site occupancy of Mg^2+^ in the order 4*f*_2_ > 4*f*_1_ > 2*b* based on Rietveld refinements [3]. Since the 4*f*_2_ site is already mixed-occupied by hafnium in the present case, an additional refinement including magnesium occupancy at the Fe(4) site was not feasible. However, as in the Mg/Zr system, it cannot be excluded that the 4*f*_2_ site is jointly occupied by Fe, Hf, and Mg. The octahedra surrounding Fe(1) (2*a*) exhibit a volume increase of approximately 3%, corresponding to roughly half of the expansion observed for the Fe(3) tetrahedra, and can therefore at least partially be attributed to neighboring substitution effects. Nevertheless, minor fractions of Mg^2+^, Hf^4+^, and/or Fe^2+^ at the octahedrally coordinated Fe(1) site cannot be ruled out. Analogous to the Mg/Zr system, the block-connecting octahedra surrounding Fe(5) (12*k*) show no significant change in volume and are therefore assumed to remain unsubstituted.

## 4. Conclusions

Mg/Zr- and Mg/Hf-co-substituted M-type ferrites were successfully synthesized using Na_2_CO_3_ as a flux and comprehensively characterized. It was observed that for nominal degrees of substitution ≥ 1, secondary phases are increasingly detected, either as perovskites or as residual starting materials. With increasing substitution levels *x*, *y* in BaFe_12−*x*−*y*_Mg*_x_M_y_*O_19_ with *M* = Zr, Hf, an increase in the lattice parameters was observed from XRD data for both substitution series, whereas the magnetic parameters *M*_S_, *M*_R_, and *H*_C_ at room temperature decrease with increasing *x*, *y*. At low temperatures, however, an increase in remanence and coercivity is observed, which can be attributed to the increasing number of magnetic domains, the weakening of superexchange interactions between them, and the limited thermal energy available.

Both Rietveld refinements of PXRD data and refinements based on SCXRD data identified Fe(4) (4*f*_2_) as the preferred site for substitution by the tetravalent metals zirconium and hafnium. Despite the significantly lower electron density of magnesium compared to iron, magnesium could not be localized from XRD data by refining site occupancies. However, insights were obtained from polyhedral volumes, which suggest that magnesium may be distributed over the Fe(3) (4*f*_1_), Fe(4) (4*f*_2_) sites, and, in the case of Mg/Hf substitution, additionally over the Fe(2) (4*e*) site. This approach clearly distinguishes the identical sites for Zr/Hf substitution as obtained from the diffraction results, but in case of Mg substitution it has to be taken with great care, since expansion of polyhedra may have a variety of reasons, for example block-level structural relaxation or local strain due to substitution of neighboring sites, or site preferences of Fe^2+^ or vacancies, which then would be difficult to discriminate from substitution by Mg. WDX analyses showed that the elements are homogeneously distributed within individual crystals and confirmed the successful incorporation of the target metals into the structure for all samples. However, within a given batch, the actual substitution level of individual crystals can vary significantly. Therefore, when interpreting the results, it is important to distinguish between measurements on single crystals and those on powder samples. In cases where parameters were taken from ground powder samples, resulting in rather average data across the sample, it is reasonable to analyze and plot those against the nominal degrees of substitution, still holding in mind a possible deviation in average composition. Data taken on individual crystals, where possible, rather benefit from applying the actual analytical composition of this crystal.

Furthermore, it was found that the tetravalent metals can be incorporated into the ferrite structure to a greater extent than magnesium, despite identical nominal substitution levels in the starting compositions. The resulting charge imbalance can be compensated for by various mechanisms. Calculations of the theoretical vacancy concentration based on WDX data provide indications of partial charge compensation via vacancy formation on transition metal sites. XANES measurements at the Fe K-edge on ground microcrystalline powder samples showed good agreement with the spectral profile of unsubstituted BaM. However, due to a lower detection limit of approximately 5%, minor fractions of Fe^2+^ cannot be excluded.

From WDX, SCXRD, and PXRD data, a maximum zirconium substitution level in Mg/Zr-substituted ferrites was determined to lie within the range 1.23(4) ≤ *y*_Zr,max_ ≤ 1.67(6). It should be noted that the lower limit is derived from WDX measurements on individual crystals and may be underestimated, as it cannot be guaranteed that the most highly substituted crystal in a batch was selected for analysis. On the other hand, this is the only method employed that enables a reliable determination of the magnesium content. The maximum observed degree of substitution for magnesium is *x*_WDX,Mg,max_ = 0.94(3), which is significantly lower than that of zirconium. The upper limit for zirconium is based on PXRD data and subsequent Rietveld refinement, thus allowing conclusions to be drawn for microcrystalline powders and polycrystalline samples. However, it should be noted that the determination of substitution levels from PXRD depends on reflection intensities, which can be influenced not only by preferred orientation but also by peak overlap with secondary phases, potentially leading to overestimation.

For Mg/Hf-substituted ferrites, the maximum degree of substitution for hafnium was found to lie within the range 1.40(5) ≤ *y*_Hf,max_ ≤ 1.47(1). The smaller variation in substitution levels obtained from powder and single-crystal XRD, as well as WDX, compared to zirconium, is likely due to the significantly larger differences in scattering power between iron and hafnium than between iron and zirconium. The lower limit again corresponds to WDX measurements, which simultaneously indicate a maximum magnesium substitution level of *x*_WDX,Mg,max_ = 1.11(4). These results suggest that, for the Mg/Hf combination, higher magnesium substitution levels are achievable than in Mg/Zr-substituted M-type ferrites. The evolution of polyhedra volumes provides evidence that this may be enabled by the distribution of magnesium over a larger number of crystallographic sites compared to Mg/Zr-substituted ferrites. This distribution over multiple sites in both substitution series also contributes to the difficulty in localizing magnesium via occupancy refinements in X-ray diffraction analyses.

In summary, the presented findings shed a differentiated light on substitution effects in co-substituted M-type ferrite systems and indicate, depending on the synthesis technique applied, that neither just reduction of iron nor equimolar substitution of differently charged ions should be considered without a closer experimental examination. Both charge balance and valence states of the constituent, as well as real composition, need to be considered to fully understand structural dependencies and physical properties of the resulting materials.

## Data Availability

The original contributions presented in this study are included in the article/Supplementary Material. Further inquiries can be directed to the corresponding author.

## Supplementary Materials

The following supporting information can be downloaded at: https://www.mdpi.com/article/10.3390/ma19122626/s1, Figures S1–S22: Rietveld refinements; Figures S23–S26: Magnetic measurements; Table S1: Unit cell parameters from Rietveld refinements; Table S2: Effective ionic radii of selected elements; Table S3: Magnetic data; Tables S4–S7: WDX data.

## Abbreviations

The following abbreviations are used in this manuscript:

a.u.	arbitrary unit
p.a.	pro analysi, for analytical purpose
BaM	BaFe_12_O_19_, unsubstituted M-type hexaferrite
PXRD	powder X-ray diffraction
SCXRD	single-crystal X-ray diffraction
SEM	scanning electron microscope
SQUID	superconducting quantum interference device
WDX	wavelength-dispersive X-ray spectroscopy
XANES	X-ray absorption near-edge structure
XAS	X-ray absorption spectroscopy
XRD	X-ray diffraction
