# Supplementary material for: Synthesis and Characterization of Magnesium Co-Substituted M-Type Ferrites BaFe12−x−yMgxMyO19 with M = Zr, Hf
Source: Materials (Basel). 2026 Jun 18;19(12):2626. doi: 10.3390/ma19122626 (PMC13304463; doi:10.3390/ma19122626)
Supplement: Supplementary file 1 [file materials-19-02626-s001.zip › materials-4331469-supplementary.pdf]

Table of Contents

1. Rietveld refinements 2
2. Effective ionic radii of selected elements... 6
3. Magnetic measurements 7
4. WDX data 8

1. Rietveld refinements

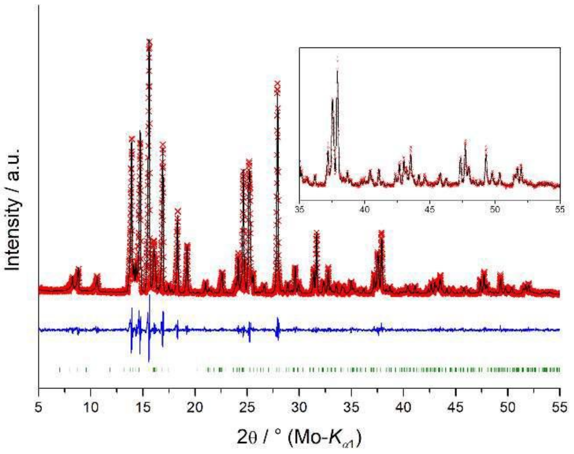

Figure S1. PXRD measurement of sample **MgZr(1)** (red) with Rietveld refinement (black), difference curve (blue), and possible Bragg positions (green). Inset: Enlarged (x2) range from 35 to 55°; gof 1.29.

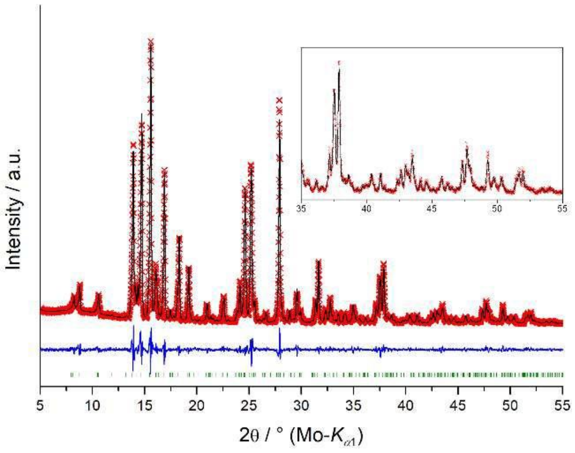

Figure S2. PXRD measurement of sample **MgZr(2)** (red) with Rietveld refinement (black), difference curve (blue), and possible Bragg positions (green). Inset: Enlarged (x2) range from 35 to 55°; gof 1.42.

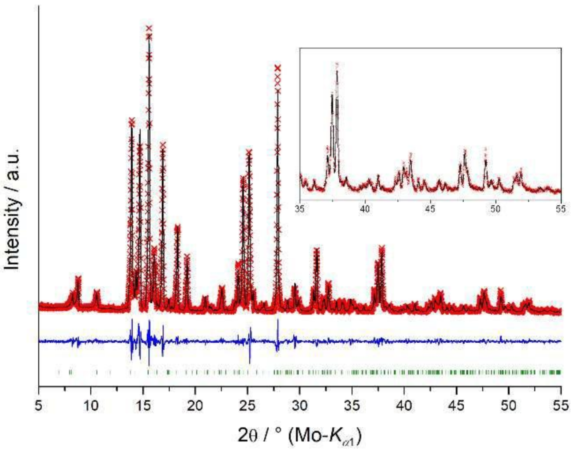

Figure S3. PXRD measurement of sample **MgZr(3)** (red) with Rietveld refinement (black), difference curve (blue), and possible Bragg positions (green). Inset: Enlarged (x2) range from 35 to 55°; gof 1.47.

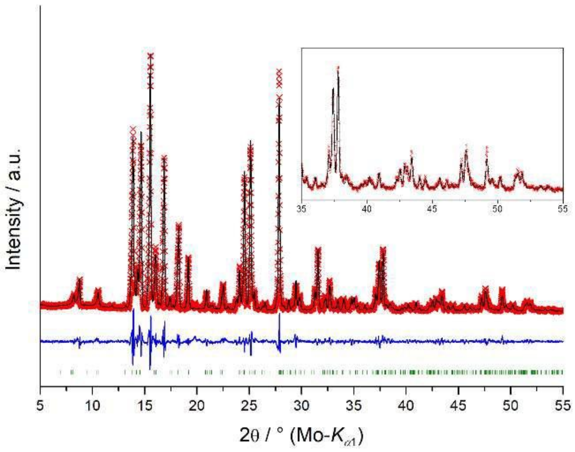

Figure S4. PXRD measurement of sample **MgZr(5)** (red) with Rietveld refinement (black), difference curve (blue), and possible Bragg positions (green). Inset: Enlarged (x2) range from 35 to 55°; gof 1.65.

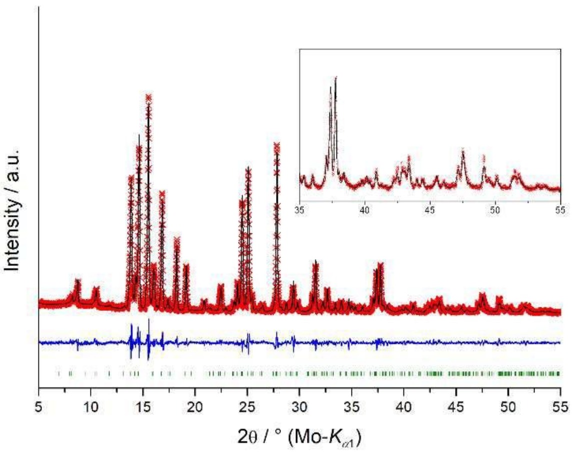

Figure S5. PXRD measurement of sample **MgZr(6)** (red) with Rietveld refinement (black), difference curve (blue), and possible Bragg positions (green). Inset: Enlarged (x2) range from 35 and 55°; gof 1.45.

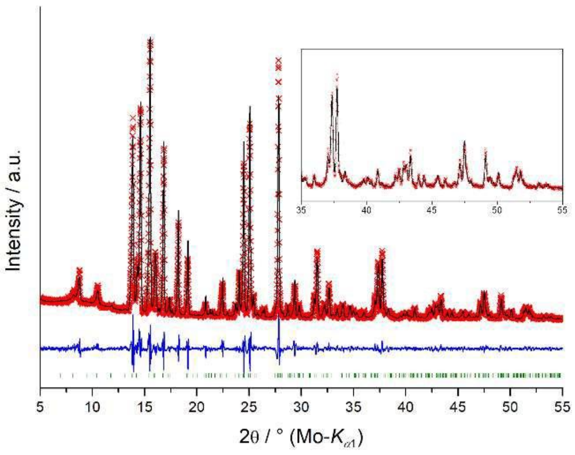

Figure S6. PXRD measurement of sample **MgZr(7)** (red) with Rietveld refinement (black), difference curve (blue), and possible Bragg positions (green). Inset: Enlarged (x2) range from 35 and 55°; gof 1.80.

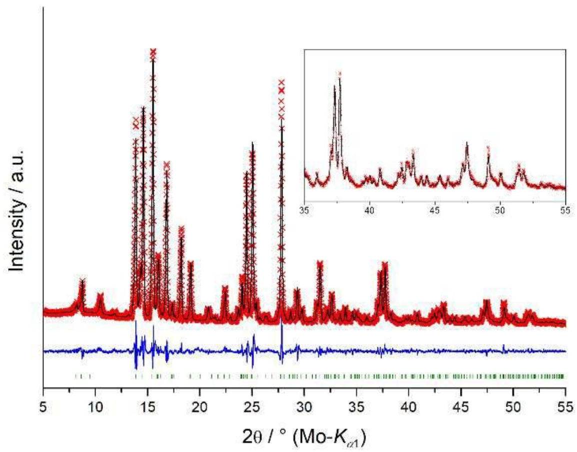

Figure S7. PXRD measurement of sample **MgZr(8)** (red) with Rietveld refinement (black), difference curve (blue), and possible Bragg positions (green). Inset: Enlarged (x2) range from 35 to 55°; gof 1.76.

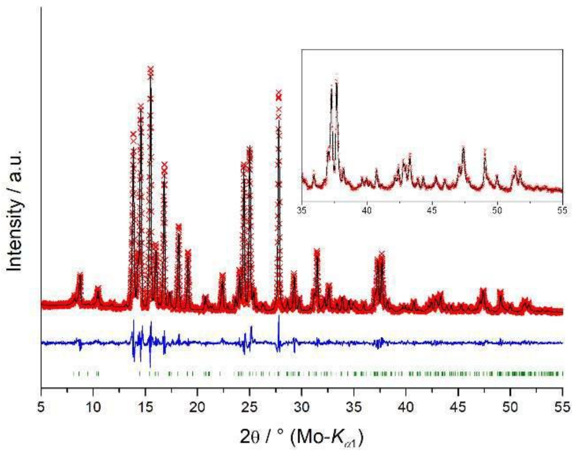

Figure S8. PXRD measurement of sample **MgZr(9)** (red) with Rietveld refinement (black), difference curve (blue), and possible Bragg positions (green). Inset: Enlarged (x2) range from 35 to 55°; gof 1.64.

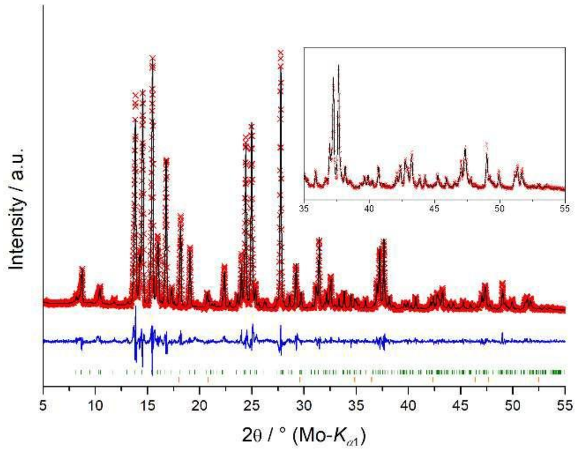

Figure S9. PXRD measurement of sample **MgZr(10)** (red) with Rietveld refinement (black), difference curve (blue), and possible Bragg positions (BaM – green, 99.69(1) wt-%; Pt – orange, 0.31(1) wt-%). Inset: Enlarged (x2) range from 35 to 55°; gof 1.84.

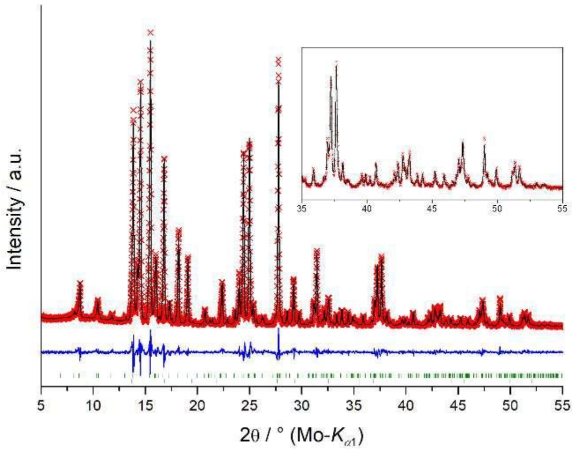

Figure S10. PXRD measurement of sample **MgZr(11)** (red) with Rietveld refinement (black), difference curve (blue), and possible Bragg positions (BaM – green, 99.61(4) wt-%; $BaZrO_3$
– dark gray, 0.39(4) wt-%). Inset: Enlarged (x2) range from 35 to 55°; gof 1.51.

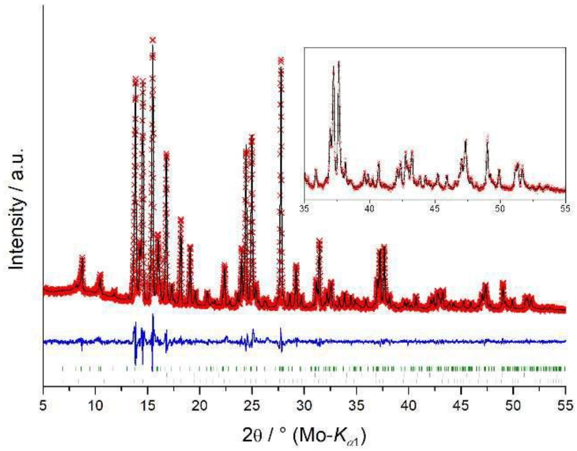

Figure S11. PXRD measurement of sample **MgZr(12)** (red) with Rietveld refinement (black), difference curve (blue), and possible Bragg positions (BaM – green, 94.99(6) wt-%; $BaZrO_3$
– dark gray, 2.01(6) wt-%; γ-Fe_2O_3 – light gray, 3.0(1) wt-%).

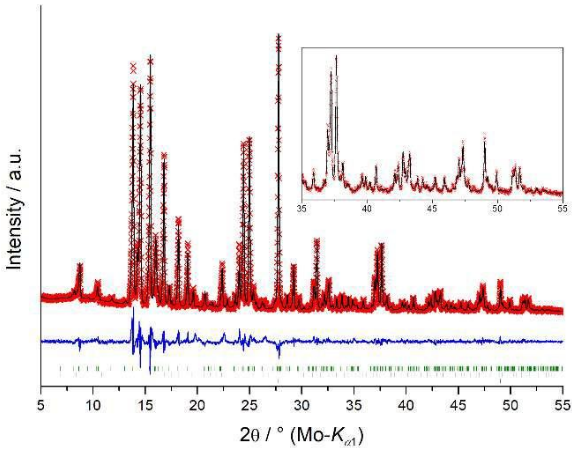

Inset: Enlarged (x2) range from 35 to 55°; gof 1.53.

Figure S12. PXRD measurement of sample **MgZr(13)** (red) with Rietveld refinement (black), difference curve (blue), and possible Bragg positions (BaM – green, 94.2(1) wt-%; *γ*-Fe_2O_3 – light gray, 2.8(1) wt-%; MgO – dark red, 3.0(1) wt-%). Inset:Enlarged (x2) range from 35 to 55°; gof 1.76.

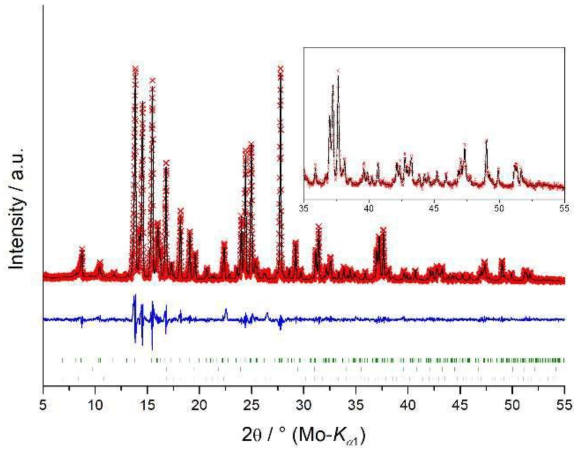

Figure S13. PXRD measurement of sample **MgZr(14)** (red) with Rietveld refinement (black), difference curve (blue), and possible Bragg positions (BaM – green, 89.8(1) wt-%; $BaZrO_3$ – dark gray, 4.35(9) wt-%; γ-Fe_2O_3 – light gray, 5.8(1) wt-%). Inset: Enlarged (x2) range from 35 to 55°; gof 1.49.

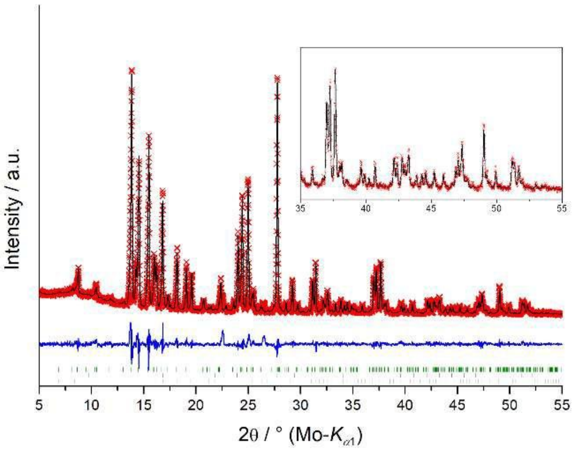

Figure S14. PXRD measurement of sample **MgZr(15)** (red) with Rietveld refinement (black), difference curve (blue), and possible Bragg positions (BaM – green, 83.9(1) wt-%; $BaZrO_3$ – dark gray, 6.3(1) wt-%; γ-Fe_2O_3 – light gray, 9.8(1) wt-%). Inset: Enlarged (x2) range from 35 to 55°; gof 1.66.

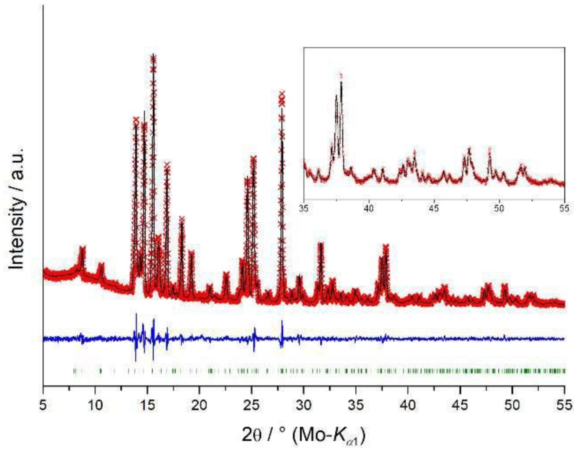

Figure S15. PXRD measurement of sample **MgHf(1)** (red) with Rietveld refinement (black), difference curve (blue), and possible Bragg positions (green). Inset: Enlarged (x2) range from 35 to 55°; gof 1.22.

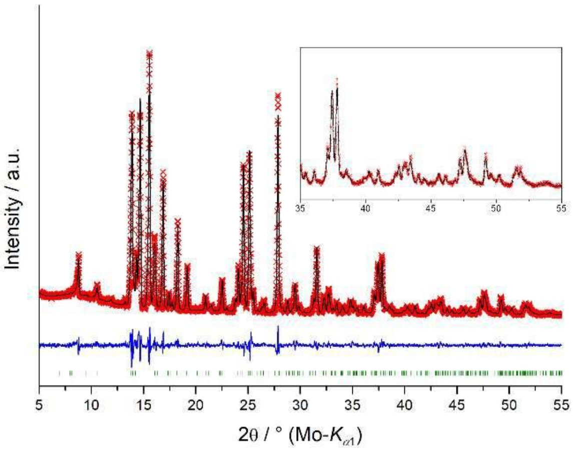

Figure S16. PXRD measurement of sample **MgHf(2)** (red) with Rietveld refinement (black), difference curve (blue), and possible Bragg positions (green). Inset: Enlarged (x2) range from 35 to 55°; gof 1.40.

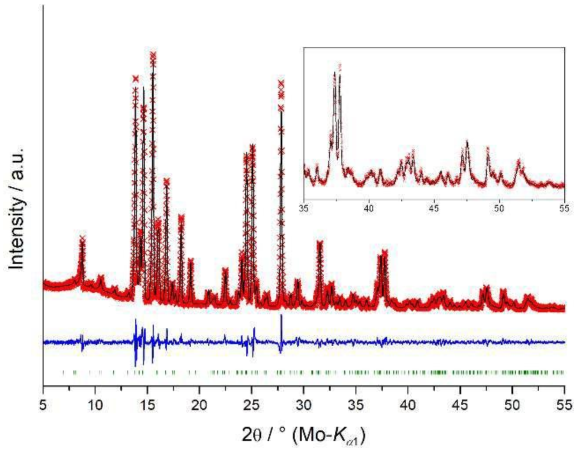

Figure S17. PXRD measurement of sample **MgHf(3)** (red) with Rietveld refinement (black), difference curve (blue), and possible Bragg positions (green). Inset: Enlarged (x2) range from 35 to 55°; gof 1.47.

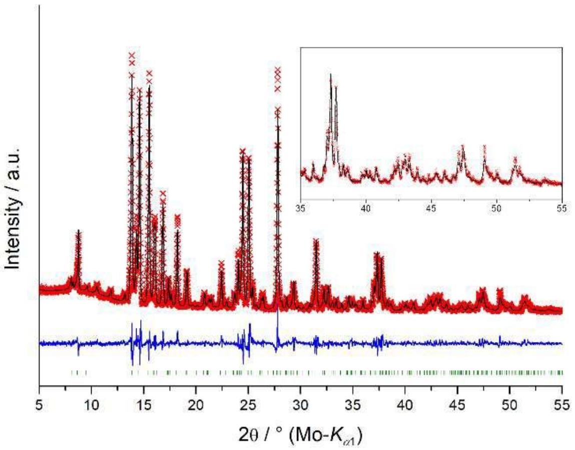

Figure S18. PXRD measurement of sample **MgHf(4)** (red) with Rietveld refinement (black), difference curve (blue), and possible Bragg positions (green). Inset: Enlarged (x2) range from 35 to 55°; gof 1.79.

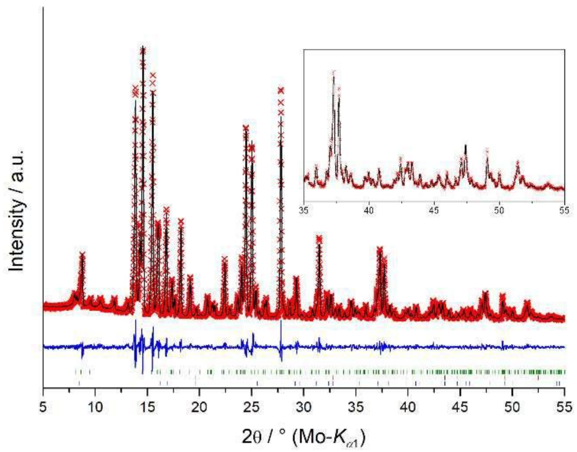

Figure S19. PXRD measurement of sample **MgHf(5)** (red) with Rietveld refinement (black), difference curve (blue), and possible Bragg positions (BaM – green, 97.6(1) wt-%; MgO – dark red, 1.5(1) wt-%; $MgFe_2O_4$ – dark blue, 0.94(9) wt-%). Inset: Enlarged (x2) range from 35 to 55°; gof 1.59.

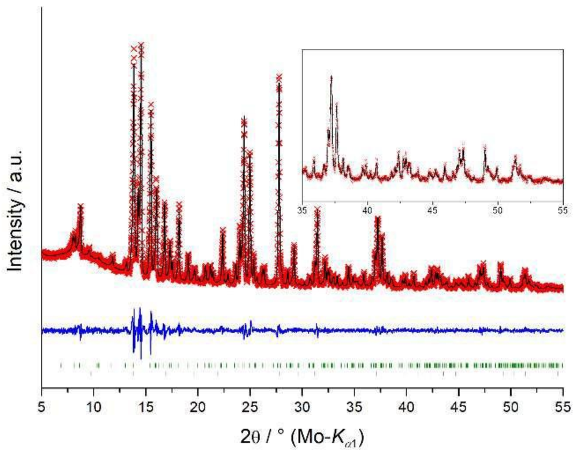

Figure S20. PXRD measurement of sample **MgHf(6)** (red) with Rietveld refinement (black), difference curve (blue), and possible Bragg positions (BaM – green, 98.11(4) wt-%; $BaHfO_3$ – dark gray, 1.89(4) wt-%). Inset: Enlarged (x2) range from 35 to 55°; gof 1.22.

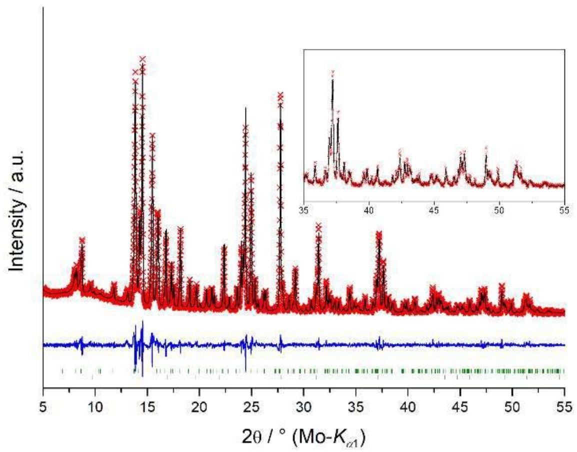

Figure S21. PXRD measurement of sample **MgHf(7)** (red) with Rietveld refinement (black), difference curve (blue), and possible Bragg positions (BaM – green, 96.38(4) wt-%; $BaHfO_3$ – dark gray, 3.62(4) wt-%). Inset: Enlarged (x2) range from 35 to 55°; gof 1.45.

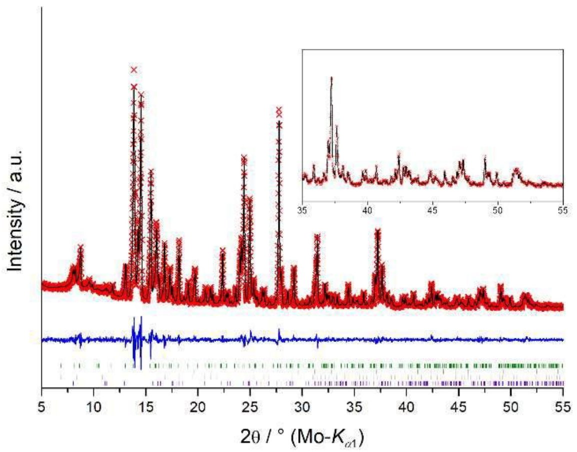

Figure S22. PXRD measurement of sample **MgHf(8)** (red) with Rietveld refinement (black), difference curve (blue), and possible Bragg positions (BaM – green, 83.7(2) wt-%; $BaHfO_3$ – dark gray, 6.31(8) wt-%; *γ*-Fe_2O_3 – light gray, 5.1(1) wt-%; HfO_2 – purple, 4.88(8) wt-%). Inset: Enlarged (x2) range from 35 to 55°; gof 1.36.

Table S1. Unit cell parameters obtained from Rietveld refinements and average degree of substitution $y_{PXRD,Zr/Hf}$ for $BaFe_{12-x-y}Mg_xZr_yO_{19}$ and $BaFe_{12-x-y}Mg_xHf_yO_{19}$.

Sample	$x_{nom} = y_{nom}$	$y_{PXRD,Zr/Hf}$	a_{PXRD} / pm	c_{PXRD} / pm	V_{PXRD} / 10^6 pm^3
**BaM**	0.00	0.00	589.820(5)	2321.28(3)	699.34(1)
**MgZr(1)**	0.10	0.23(3)	590.23(1)	2325.47(6)	701.60(3)
**MgZr(2)**	0.20	0.28(3)	590.46(1)	2328.27(6)	702.99(3)
**MgZr(3)**	0.30	0.55(3)	590.98(1)	2333.27(7)	705.75(3)
**MgZr(4)**	0.40	0.62(4)	591.14(1)	2335.5(1)	706.79(4)
**MgZr(5)**	0.50	0.74(4)	591.48(1)	2339.15(9)	708.72(4)
**MgZr(6)**	0.60	0.91(4)	592.09(1)	2343.77(8)	711.57(4)
**MgZr(7)**	0.70	0.98(5)	592.26(1)	2347.53(9)	713.12(4)
**MgZr(8)**	0.80	1.13(4)	592.48(1)	2353.42(9)	715.44(4)
**MgZr(9)**	0.90	1.30(4)	593.01(1)	2358.9(1)	718.42(4)
**MgZr(10)**	1.00	1.43(5)	593.47(1)	2361.39(8)	720.27(4)
**MgZr(11)**	1.10	1.34(3)	593.566(9)	2363.49(6)	721.15(3)
**MgZr(12)**	1.20	1.45(4)	593.57(1)	2363.25(8)	721.07(3)
**MgZr(13)**	1.30	1.41(5)	593.45(1)	2363.46(8)	720.86(3)
**MgZr(14)**	1.40	1.67(6)	593.53(1)	2363.81(9)	721.16(4)
**MgZr(15)**	1.50	1.65(6)	593.39(1)	2362.85(9)	720.53(4)
**MgHf(1)**	0.20	0.218(8)	590.58(1)	2328.76(8)	703.42(3)
**MgHf(2)**	0.40	0.476(9)	591.43(1)	2336.09(8)	707.65(3)
**MgHf(3)**	0.60	0.69(1)	592.12(1)	2343.08(9)	711.43(4)
**MgHf(4)**	0.80	0.92(1)	592.89(1)	2352.7(1)	716.24(5)
**MgHf(5)**	1.00	1.040(8)	592.87(1)	2355.99(7)	717.18(3)
**MgHf(6)**	1.20	1.31(1)	593.51(1)	2364.90(7)	721.43(3)
**MgHf(7)**	1.40	1.45(1)	593.826(9)	2367.06(5)	722.87(3)
**MgHf(8)**	1.60	1.44(1)	593.46(1)	2365.58(7)	721.53(3)

2. Effective ionic radii of selected elements

Table S2. Effective ionic radii depending on their coordination number, *CN,* according to Shannon [1], of the relevant likely present metal ions.

Ion	*CN* (spin state)	Effective ionic radius/pm
Fe^{2+}	IV (high spin)	63
	VI (high spin)	78
Fe^{3+}	IV (high spin)	49
	V	58
	VI (high spin)	64.5
Mg^{2+}	IV	57
	V	66
	VI	72
Zr^{4+}	VI	72
Hf^{4+}	VI	71

3. Magnetic measurements

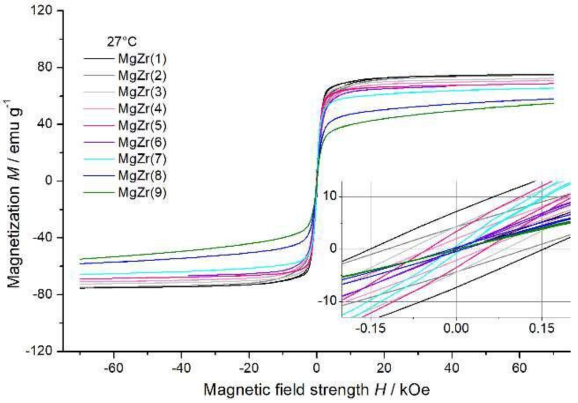

Figure S23. Hysteresis curve of samples **MgZr(1)** – **MgZr(9)** measured at 27 °C. Inset: Enlargement of the area around the origin.

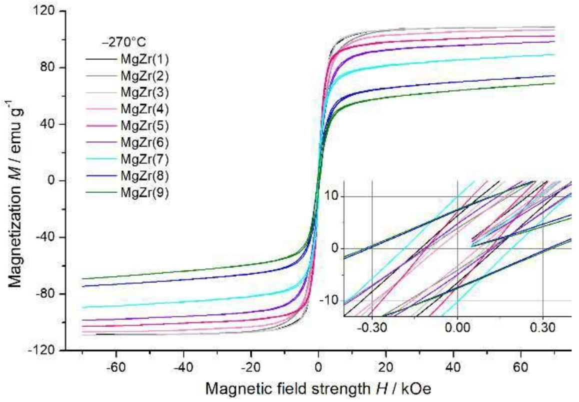

Figure S24. Hysteresis curve of samples **MgZr(1)** – **MgZr(9)** measured at −270 °C. Inset: Enlargement of the area around the origin.

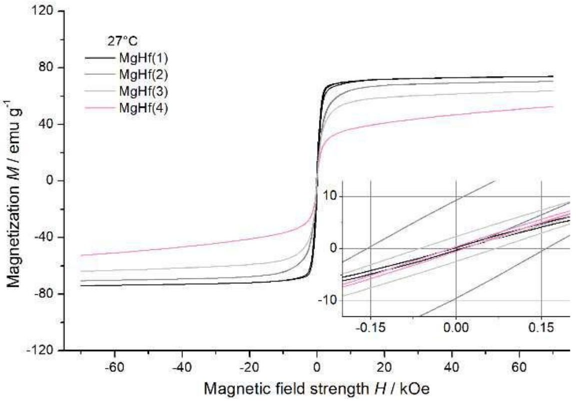

Figure S25. Hysteresis curve of samples **MgHf(1)** – **MgHf(4)** measured at 27 °C. Inset: Enlargement of the area around the origin.

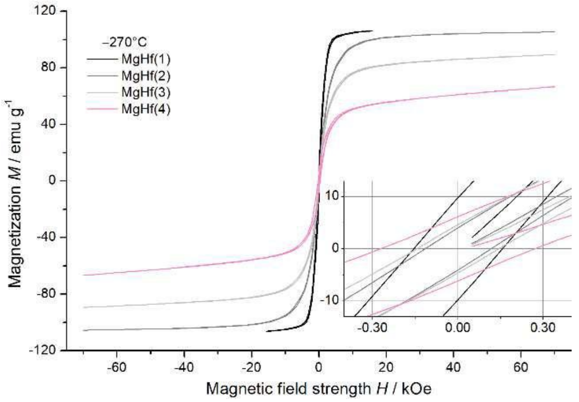

Figure S26. Hysteresis curve of samples **MgHf(1)** – **MgHf(4)** measured at −270 °C. Inset: Enlargement of the area around the origin.

Table S3. Derived magnetic data for all single-phase samples at –270 °C and 27 °C, corresponding to Figures S21 – S24.

Sample	$x_{nom} = y_{nom}$	–270 °C			27 °C		
		H_C / Oe	M_R / emu g^{-1}	M_S / emu g^{-1}	H_C / Oe	M_R / emu g^{-1}	M_S / emu g^{-1}
BaM	0.00	103.07	4.50	104.48	119.25	5.09	74.43
MgZr(1)	0.10	131.96	6.44	108.66	152.31	7.24	75,3579
MgZr(2)	0.20	110.50	4.04	108.76	122.55	4.28	74,7743
MgZr(3)	0.30	94.36	4.96	109.06	83.95	4.35	72,4373
MgZr(4)	0.40	71.66	3.21	106.73	46.07	2.07	70,8982
MgZr(5)	0.50	111.85	7.17	102.72	50.58	3.44	68,7731
MgZr(6)	0.60	119.71	4.88	98.44	2.63	0.13	66,574
MgZr(7)	0.70	187.72	10.02	89.38	8.82	0.69	65,6131
MgZr(8)	0.80	315.67	7.37	74.42	10.85	0.37	58,0839
MgZr(9)	0.90	328.16	7.56	69.12	0.90	0.03	54,9285
MgHf(1)	0.20	155.88	9.85	106.17	158.90	9.39	74.00
MgHf(2)	0.40	115.87	4.06	105.56	66.73	2.38	70.48
MgHf(3)	0.60	144.62	4.59	89.36	11.02	0.39	63.80
MgHf(4)	0.80	271.98	6.17	66.69	7.41	0.25	52.62

4. WDX data

Table S4. Average mass fractions $\overline{w}$ of the elements obtained from WDX measurements and their sum for compounds of the composition $BaFe_{12-x-y}Mg_xZr_yO_{19}$. The oxygen content was calculated under the assumption that the elements are present in the oxidation states Ba^{2+}, Fe^{3+}, Mg^{2+}, and Zr^{4+}. Samples labeled with the index “sc” were additionally investigated by single-crystal X-ray diffraction; this does not applyto crystals labeled with the index “r”.

Sample	w_{Ba} / wt-%	w_{Fe} / wt-%	w_{Mg} / wt-%	w_{Zr} / wt-%	w_O / wt-%	Total / wt-%
$MgZr(1)_{sc1}$	13.0(3)	59(1)	0.10(3)	0.73(8)	27.31	100.4
$MgZr(2)_{sc1}$	12.9(3)	62(1)	0.05(2)	0.01(3)	28.05	102.7
$MgZr(3)_{sc1}$	11.9(3)	50.4(9)	0.43(4)	3.5(1)	24.55	90.8
$MgZr(4)_{sc1}$	11.8(3)	60(1)	0.91(5)	1.8(1)	28.18	102.2
$MgZr(4)_{sc2}$	12.5(3)	57(1)	0.66(4)	4.2(1)	27.80	102.0
$MgZr(5)_{sc1}$	12.8(3)	56(1)	0.49(3)	5.1(2)	27.48	101.4
$MgZr(6)_{sc1}$	12.1(3)	56(1)	1.22(5)	2.0(2)	27.86	102.0
$MgZr(7)_{sc1}$	11.7(3)	46.8(9)	1.74(6)	7.8(2)	25.32	93.2
$MgZr(8)_{sc1}$	11.6(3)	55(1)	0.35(3)	2.0(1)	25.79	94.4
$MgZr(9)_{sc1}$	12.5(3)	50.6(9)	1.79(6)	9.0(2)	27.54	101.4
$MgZr(9)_{r1}$	12.5(3)	51.9(9)	1.86(6)	8.7(2)	28.04	103.0
$MgZr(10)_{sc1}$	11.1(3)	52.1(9)	1.57(6)	7.9(2)	27.47	100.1
$MgZr(11)_{r1}$	11.7(3)	54(1)	1.36(5)	7.5(2)	28.11	102.7
$MgZr(12)_{sc1}$	12.0(3)	49.2(7)	2.03(6)	9.8(3)	27.30	100.3
$MgZr(13)_{sc1}$	12.4(3)	50.6(9)	2.09(6)	9.6(3)	27.93	102.6
$MgZr(13)_{r1}$	11.5(3)	46.1(9)	1.52(5)	9.4(3)	25.45	94.0
$MgZr(14)_{sc1}$	10.3(2)	51.8(9)	0.63(4)	6.4(2)	26.11	95.2
$MgZr(15)_{sc1}$	12.2(3)	50.7(9)	1.77(6)	9.3(3)	27.62	101.6

Table S5. Average mass fractions $\overline{w}$ of the elements obtained from WDX measurements and their sum for compounds of the composition $BaFe_{12-x-y}Mg_xHf_yO_{19}$. The oxygen content was calculated under the assumption that the elements are present in the oxidation states Ba^{2+}, Fe^{3+}, Mg^{2+}, and Hf^{4+}. For some measurements, the samples were coated with elemental gold to ensure electrical conductivity; in these cases,the mass fraction of gold is additionally reported. Samples labeled with the index “sc” were additionally investigated by single-crystal X-ray diffraction; this does not apply to crystals labeled with the index “r”.

Sample	w_{Ba} / wt-%	w_{Fe} / wt-%	w_{Mg} / wt-%	w_{Zr} / wt-%	w_O / wt-%	w_{Au} / wt-%	Total / wt-%
$MgHf(1)_{sc1}$	12.2(3)	54.6(6)	0.25(3)	3.7(3)	25.71	—	96.4
$MgHf(1)_{r1}$	11.7(3)	56.2(8)	0.25(3)	4.1(2)	26.40	3.5(3)	102.1
$MgHf(1)_{r2}$	11.5(3)	52.5(7)	0.45(4)	7.3(2)	25.48	3.7(3)	100.9
$MgHf(2)_{r1}$	11.4(3)	52.9(7)	0.51(4)	7.2(2)	25.70	3.5(3)	101.2
$MgHf(2)_{r2}$	11.2(3)	56.8(8)	0.37(4)	2.6(2)	26.40	3.4(3)	100.7
$MgHf(2)_{r3}$	11.5(3)	52.7(7)	0.38(4)	7.3(2)	25.57	3.5(3)	101.0
$MgHf(3)_{sc1}$	11.8(3)	50.5(5)	0.60(4)	9.2(4)	25.10	—	97.1
$MgHf(3)_{r1}$	10.7(3)	50.6(7)	1.19(6)	8.7(3)	25.32	3.6(3)	100.1
$MgHf(4)_{sc1}$	11.2(3)	47.7(5)	0.63(4)	9.4(4)	23.89	—	92.8
$MgHf(4)_{r1}$	11.1(3)	46.4(9)	1.47(6)	15.8(4)	25.02	—	99.8
$MgHf(4)_{r2}$	11.1(3)	49.5(9)	1.24(6)	14.5(3)	25.98	—	102.3
$MgHf(4)_{r3}$	11.0(3)	49.9(9)	1.03(5)	13.5(3)	25.83	—	101.3
$MgHf(5)_{sc1}$	10.9(3)	43.1(5)	0.94(4)	15.6(5)	23.21	—	93.7
$MgHf(5)_{r1}$	11.6(3)	50.7(9)	0.82(5)	12.5(3)	25.92	—	101.5
$MgHf(5)_{r2}$	10.1(2)	45.8(8)	1.51(6)	15.3(4)	24.59	—	97.3
$MgHf(5)_{r3}$	10.8(3)	47.6(9)	1.33(6)	16.2(4)	25.48	—	101.3
$MgHf(6)_{sc1}$	9.5(2)	47.2(7)	1.77(6)	15.6(4)	25.35	—	99.4
$MgHf(6)_{sc2}$	10.3(2)	44.3(6)	1.27(5)	16.9(4)	24.11	—	96.9
$MgHf(6)_{sc3}$	10.1(2)	48.0(7)	1.15(5)	14.7(4)	25.21	—	99.2
$MgHf(6)_{r1}$	10.4(3)	46.6(9)	1.34(6)	16.6(4)	25.10	—	100.0
$MgHf(6)_{r2}$	10.5(3)	45.0(8)	1.39(6)	18.0(4)	24.69	—	99.5
$MgHf(6)_{r3}$	10.3(3)	44.3(8)	1.53(6)	17.3(4)	24.32	—	97.7
$MgHf(7)_{sc1}$	10.2(2)	40.0(4)	2.06(7)	19.0(6)	23.12	—	94.3
$MgHf(7)_{sc2}$	10.3(2)	43.6(6)	1.85(6)	19.2(5)	24.60	—	99.6
$MgHf(7)_{r1}$	9.9(2)	42.8(6)	1.82(6)	18.5(4)	24.05	3.8(3)	100.8
$MgHf(8)_{sc1}$	9.8(2)	42.3(5)	1.70(6)	16.4(6)	23.39	—	93.6
$MgHf(8)_{r1}$	9.5(2)	43.3(6)	1.56(6)	17.7(4)	23.92	3.8(3)	99.7

Table S6. Calculated fractions of iron (Fe_{WDX}), magnesium ($x_{WDX,Mg}$), and zirconium ($y_{WDX,Zr}$) from WDX measurements for the investigated crystals with the general composition $BaFe_{12-x-y}Mg_xZr_yO_{19}$, as well as the calculated vacancy concentration $\square_{WDX,Mg/Zr}$. Samples labeled with the index "sc" were additionally investigated by single-crystal X-ray diffraction; this does not apply to crystals labeled with the index "r".

Crystal	$x_{nom} = y_{nom}$	Fe_{WDX}	$x_{WDX,Mg}$	$y_{WDX,Zr}$	$\square_{WDX,Mg/Zr}$
$MgZr(1)_{sc1}$	0.10	11.82(21)	0.04(1)	0.09(1)	0.05
$MgZr(2)_{sc1}$	0.20	11.97(21)	0.02(1)	0.001(3)	0.009
$MgZr(3)_{sc1}$	0.30	11.17(21)	0.22(2)	0.48(2)	0.13
$MgZr(4)_{sc1}$	0.40	11.50(21)	0.40(2)	0.21(1)	−0.11
$MgZr(4)_{sc2}$	0.40	11.13(20)	0.30(2)	0.51(2)	0.06
$MgZr(5)_{sc1}$	0.50	11.01(20)	0.22(2)	0.62(3)	0.15
$MgZr(6)_{sc1}$	0.60	10.91(20)	0.39(2)	0.65(3)	0.05
$MgZr(7)_{sc1}$	0.70	10.05(20)	0.86(3)	1.02(3)	0.07
$MgZr(8)_{sc1}$	0.80	11.54(21)	0.17(2)	0.26(2)	0.03
$MgZr(9)_{sc1}$	0.90	10.00(19)	0.81(3)	1.09(4)	0.10
$MgZr(9)_{r1}$	0.90	10.07(19)	0.83(3)	1.03(3)	0.07
$MgZr(10)_{sc1}$	1.00	10.32(19)	0.71(3)	0.96(3)	0.01
$MgZr(11)_{r1}$	1.10	10.46(19)	0.61(2)	0.89(3)	0.04
$MgZr(12)_{sc1}$	1.20	9.80(15)	0.93(3)	1.20(4)	0.07
$MgZr(13)_{sc1}$	1.30	9.88(19)	0.94(3)	1.15(4)	0.05
$MgZr(13)_{r1}$	1.30	9.86(19)	0.72(2)	1.23(4)	0.17
$MgZr(14)_{sc1}$	1.40	10.79(20)	0.30(2)	0.82(3)	0.09
$MgZr(15)_{sc1}$	1.50	9.98(19)	0.80(3)	1.12(4)	0.10

Table S7. Calculated fractions of iron (Fe_{WDX}), magnesium ($x_{WDX,Mg}$), and zirconium ($y_{WDX,Hf}$) from WDX measurements for the investigated crystals with the general composition $BaFe_{12-x-y}Mg_xHf_yO_{19}$, as well as the calculated vacancy concentration $\square_{WDX,Mg/Hf}$. Samples labeled with the index "sc" were additionally investigated by single-crystal X-ray diffraction; this does not apply to crystals labeled with the index "r".

Crystal	$x_{nom} = y_{nom}$	Fe_{WDX}	$x_{WDX,Mg}$	$y_{WDX,Hf}$	$\square_{WDX,Mg/Hf}$
$MgHf(1)_{sc1}$	0.20	11.56(13)	0.12(1)	0.25(2)	0.07
$MgHf(1)_{r1}$	0.20	11.59(17)	0.12(1)	0.26(2)	0.03
$MgHf(1)_{r2}$	0.20	11.21(17)	0.22(2)	0.48(2)	0.09
$MgHf(2)_{r1}$	0.40	11.70(17)	0.18(2)	0.17(1)	−0.05
$MgHf(2)_{r2}$	0.40	11.21(18)	0.25(2)	0.48(2)	0.06
$MgHf(2)_{r3}$	0.40	11.23(17)	0.19(2)	0.49(2)	0.09
$MgHf(3)_{sc1}$	0.60	10.94(13)	0.30(2)	0.62(3)	0.14
$MgHf(3)_{r1}$	0.60	10.87(16)	0.58(3)	0.59(2)	−0.04
$MgHf(4)_{sc1}$	0.80	10.85(13)	0.33(2)	0.68(3)	0.14
$MgHf(4)_{r1}$	0.80	10.52(20)	0.50(2)	0.89(2)	0.09
$MgHf(4)_{r2}$	0.80	10.37(20)	0.60(3)	0.95(3)	0.08
$MgHf(4)_{r3}$	0.80	10.09(20)	0.74(3)	1.07(3)	0.10
$MgHf(5)_{sc1}$	1.00	10.12(12)	0.50(2)	1.14(4)	0.24
$MgHf(5)_{r1}$	1.00	10.65(20)	0.39(2)	0.82(2)	0.14
$MgHf(5)_{r2}$	1.00	10.14(20)	0.77(3)	1.06(3)	0.03
$MgHf(5)_{r3}$	1.00	10.17(20)	0.65(3)	1.08(3)	0.10
$MgHf(6)_{sc1}$	1.20	10.14(16)	0.87(3)	1.05(3)	−0.06
$MgHf(6)_{sc2}$	1.20	10.01(15)	0.66(3)	1.19(3)	0.14
$MgHf(6)_{sc3}$	1.20	10.36(16)	0.57(2)	0.89(3)	0.18
$MgHf(6)_{r1}$	1.20	10.11(20)	0.67(3)	1.13(3)	0.09
$MgHf(6)_{r2}$	1.20	9.90(19)	0.79(3)	1.21(3)	0.10
$MgHf(6)_{r3}$	1.20	9.92(19)	0.71(3)	1.24(3)	0.13
$MgHf(7)_{sc1}$	1.40	9.41(11)	1.11(4)	1.40(5)	0.08
$MgHf(7)_{sc2}$	1.40	9.65(15)	0.94(3)	1.33(3)	0.08
$MgHf(7)_{r1}$	1.40	9.68(15)	0.95(3)	1.31(3)	0.06
$MgHf(8)_{sc1}$	1.60	9.85(12)	0.91(3)	1.20(4)	0.04
$MgHf(8)_{r1}$	1.60	9.86(15)	0.81(3)	1.26(3)	0.07

References

1. Shannon, R.D. Revised effective ionic radii and systematic studies of interatomic distances in halides and chalcogenides. *Acta Crystallogr. A* **1976**, *32*, 751–767, doi:10.1107/S0567739476001551.
